# Dynamics of PBMC gene expression in hepatitis C virus genotype 1-infected patients during combined peginterferon/ribavirin therapy

**DOI:** 10.18632/oncotarget.11348

**Published:** 2016-08-17

**Authors:** Ming-Ying Lu, Ching-I Huang, Ming-Yen Hsieh, Tusty-Juan Hsieh, Edward Hsi, Pei-Chien Tsai, Yi-Shan Tsai, Ching-Chih Lin, Meng-Hsuan Hsieh, Po-Cheng Liang, Yi-Hung Lin, Nai-Jen Hou, Ming-Lun Yeh, Chung-Feng Huang, Zu-Yau Lin, Shinn-Cherng Chen, Jee-Fu Huang, Wan-Long Chuang, Chia-Yen Dai, Ming-Lung Yu

**Affiliations:** ^1^ Hepatobiliary Division, Department of Internal Medicine and Hepatitis Center, Kaohsiung Medical University Hospital, Kaohsiung, Taiwan; ^2^ Faculty of Internal Medicine, College of Medicine, and Graduate Institute of Clinical Medicine, and Center for Infectious Disease and Cancer Research, Kaohsiung Medical University, Kaohsiung, Taiwan; ^3^ Institute of Biomedical Sciences, National Sun Yat-Sen University, Kaohsiung, Taiwan; ^4^ Department of Internal Medicine, Kaohsiung Municipal Hsiao-Kang Hospital, Kaohsiung Medical University, Kaohsiung, Taiwan; ^5^ Department of Occupational Medicine, Kaohsiung Municipal Ta-Tung Hospital, Kaohsiung Medical University, Kaohsiung, Taiwan; ^6^ Department of Preventive Medicine, Kaohsiung Medical University Hospital, Kaohsiung, Taiwan; ^7^ Graduate Institute of Medicine, College of Medicine, Kaohsiung Medical University, Kaohsiung, Taiwan; ^8^ Department of Genome Medicine, College of Medicine, Kaohsiung Medical University, Kaohsiung, Taiwan; ^9^ Department of Medical Research, Kaohsiung Medical University Hospital, Kaohsiung, Taiwan; ^10^ Liver Center, Division of Gastroenterology, Massachusetts General Hospital, Harvard Medical School, Boston, MA, USA

**Keywords:** hepatitis C, interferon, sustained virologic response

## Abstract

Hepatitis C virus (HCV) can replicate in peripheral blood mononuclear cells (PBMCs), which can produce interferon to defend against virus infection. We hypothesized that dynamic gene expression in PBMCs might impact the treatment efficacy of peginterferon/ribavirin in HCV patients. PBMCs were collected at baseline, 1^st^ week and 4^th^ week of treatment from 27 chronic HCV-1 patients with 48-week peginterferon/ribavirin therapy (screening dataset *n* = 7; validation dataset *n* = 20). A sustained virologic response (SVR) was defined as undetectable HCV RNA throughout the 24 weeks after end-of-treatment. A complete early virologic response (cEVR) was defined as negative HCV RNA at treatment week 12. Forty-three differentially expressed genes identified by Affymetrix microarray were validated by quantitative polymerase chain reaction. Thirteen genes at week 1 and 24 genes at week 4 were upregulated in the SVR group compared with the non-SVR group. We selected 8 target genes (RSAD2, LOC26010, HERC5, HERC6, IFI44, SERPING1, IFITM3, and DDX60) at week 1 as the major components of the predictive model. This predictive model reliably stratified the responders and non-responders at week 1 (AUC = 0.89, *p* = 0.007 for SVR; AUC = 0.95, *p* = 0.003 for cEVR), especially among patients carrying the IL28B rs8099917 TT genotype (AUC = 0.89, *p* = 0.02 for SVR; AUC = 1.0, *p* = 0.008 for cEVR). The performance of this predictive model was superior to traditional predictors, including the rapid virologic response, viral load and IL28B genotype.

## INTRODUCTION

Hepatitis C virus (HCV) affects 180 million people worldwide and is the major cause of liver cirrhosis [1]. Approximately 1%–4% of cirrhotic patients may progress to the development of hepatocellular carcinoma [2]. There are six HCV genotypes and more than 50 subtypes worldwide [3]. The most common HCV genotypes in Taiwan are genotypes 1b, 2a, 2b and 3a [4]. Combined peginterferon (pegIFN) and ribavirin therapy results in a suboptimal sustained virologic response (SVR) and intolerable adverse effects [5]. Administration of a 48-week pegIFN/ribavirin regimen can achieve a sustained virologic response (SVR) rate of 40%–70% in HCV-1 infected patients. In contrast, the SVR rate is approximately 90% for HCV-2 or HCV-3 infected patients treated with pegIFN/ribavirin therapy for 24 weeks [6]. New generation direct acting antiviral agents (DAAs) have fewer side effects and substantially improve the SVR rates up to 90% for HCV-1 [7–9]. However, the high cost of DAAs limits their clinical application. Thus, the pegIFN/ribavirin regimen remains a mainstay of HCV therapy in developing countries.

Many viral and host factors are responsible for the pathogenesis of HCV infection [10, 11]. HCV genotypes [12], viral loads [13], IL-28B polymorphisms [14], and a rapid virologic response (RVR) [15] have been proposed as important predictors for the treatment outcome of pegIFN/ribavirin. However, the reason why a substantial proportion of patients fail PegIFN/ribavirin therapy remains unclear. Although HCV primarily replicates in hepatocytes, there is evidence that peripheral blood mononuclear cells (PBMCs) can serve as a suitable site for HCV extrahepatic replication [16, 17]. PBMCs are also potent producers of interferon to defend against virus invasion [18]. Therefore, we hypothesized that responders and non-responders might have different PBMC gene expression patterns during pegIFN/ribavirin therapy.

Understanding the molecular basis of the pegIFN/ribavirin therapy response for HCV infection is essential for personalized medicine. We explored the dynamic gene expression profiles of PBMCs collected from HCV-1-infected patients undergoing pegIFN/ribavirin therapy. We aimed to establish a genetic model to predict the treatment response to pegIFN/ribavirin therapy. Furthermore, we examined the possible molecular mechanisms underlying interferon therapy.

## RESULTS

### Baseline characteristics

The demographic characteristics of the study subjects were shown in Table 1. A total of 27 HCV-1 patients (screening dataset *n* = 7; validation dataset *n* = 20) treated with 48 weeks of pegIFN/ribavirin were recruited in this study. Four (57.1%) patients in the screening study and 12 (60.0%) patients in the validation study achieved a sustained virologic response (SVR). The frequency of the favorable IL28B rs8099917 TT genotype in the overall cases was 83.3%. All patients carrying the unfavorable IL28B rs8099917 GT genotype (*n* = 4) failed to achieve SVR. There was no significant difference in the gender, age, GOT, GPT, viral load and IL28B rs8099917 genotype in the screening and validation datasets. The baseline demographic characteristics were comparable between the screening and validation datasets.

**Table 1:** Baseline demographics of HCV-1 patients

	Screening			Validation			Screening vs. Validation *p*-value	
	SVR	non-SVR	*p*-value	SVR	non-SVR	*p*-value	SVR	non-SVR
n	4	3		12	8		-	-
Age (years, mean ± SD)	49.0 ± 17.6	49.7 ± 8.6	0.955	42.4 ± 10.6	42.8 ± 12.8	0.950	0.516	0.416
Sex (M/F)	4/0	2/1	0.429	8/4	7/1	0.603	0.376	0.491
GOT (IU/L, mean ± SD)	144.8 ± 178.2	127.0 ± 36.5	0.875	68.0 ± 36.8	62.1 ± 48.5	0.762	0.454	0.067
GPT (IU/L, mean ± SD)	74.5 ± 25.2	171.7 ± 92.8	0.207	120.4 ± 86.1	97.8 ± 67.2	0.539	0.320	0.172
HCV RNA (log IU/ml)	5.33 ± 1.34	6.44 ± 0.75	0.259	5.03 ± 1.02	5.69 ± 0.90	0.154	0.218	0.147
IL28B rs8099917 TT	4 (100.0%)	2 (66.7%)	0.429	10 (100.0%)	4 (57.1%)	0.051	-	1.000
GT	0 (0.0%)	1 (33.3%)		0 (0.0%)	3 (42.9%)			

p.s. SVR: sustained virologic response; non-SVR: non-sustained virologic response.

### Microarray analysis

Forty-three differentially expressed genes were obtained from the microarray profiling of the PBMC samples (SVR *n* = 4; non-SVR *n* = 3) during pegIFN/ribavirin therapy (Supplementary Table S1). Among the 43 differentially expressed genes, 16 genes at week 1 and 23 genes at week 4 were significantly upregulated in the SVR group compared with the non-SVR group. In contrast, four genes at week 4 were significantly downregulated in the SVR group compared with the non-SVR group. A total of 43 genes were candidates for real-time PCR validation.

The gene ontology (GO) analysis showed the functional classification of these 43 differentially expressed genes. Catalytic (32.6%) and binding activity (30.2%) were the represented biological functions. Among the genes with catalytic activity, 7 genes (38.9%) had hydrolase activity, 6 genes (33.3%) had transferase activity, and 2 genes (11.1%) had ligase activity. Among the genes with binding activity, 7 genes (53.8%) had nucleic acid binding activity and 6 genes (46.2%) had protein binding activity (Supplementary Figure S1).

### Real-time PCR validation

The PBMC gene expression signature at baseline was not significantly different between the responders and non-responders. Thirteen genes at week 1 (Supplementary Figure S2) and 24 genes at week 4 (Supplementary Figure S3) were significantly upregulated in the SVR group compared with the non-SVR group (Supplementary Table S2). Twenty-five genes at week 1 and 27 genes at week 4 were significantly upregulated in the cEVR group compared to the non-cEVR group (Supplementary Table S3). None of the significantly downregulated genes at baseline, week 1 and week 4 were confirmed by quantitative PCR.

### Gene scores

We attempted to establish a genetic model to predict the treatment outcome of pegIFN/ribavirin for HCV-1 patients. We speculated that the genes correlated with the treatment response might be persistently expressed during pegIFN/ribavirin therapy. The target genes of the predictive model met the following criteria: (1) the differentially expressed genes between the SVR and non-SVR groups (fold change > 1.7 and *p-value* < 0.05) were present at both weeks 1 and 4. (2) The minimal number of target genes was favored under the premise of the precise prediction of the treatment outcome. We selected 8 target genes (RSAD2, LOC26010, HERC5, HERC6, IFI44, SERPING1, IFITM3, and DDX60) that were expressed at both weeks 1 and 4 as the major components of the predictive model. The cellular locations and functions of these target genes were listed in Table 2. The gene score was defined as the cumulative fold change of the candidate genes [i.e., gene score = sum of fold change (RSAD2 + LOC26010 + HERC5 + HERC6 + IFI44 + SERPING1 + IFITM3 + DDX60)]. The expression of the candidate genes was normalized to the GADPH endogenous control. The relative expression of the candidate genes was compared with the mean dCT of the non-SVR group.

**Table 2:** List of gene panel

Symbol	Chr.	Location	Gene name	Function
LOC26010	2q33.1	Nucleus	viral DNA polymerase-transactivated protein 6	Involved in ribosome biogenesis and translational control in response to oxidative stress.
IFI44	1p31.1	Cytoplasm	interferon-induced protein 44	This protein aggregates to form microtubular structures
RSAD2	2p25.2	Cytoplasm	radical S-adenosyl methionine domain containing 2	Involved in antiviral defense. May impair virus budding by disrupting lipid rafts at the plasma membrane.
HERC5	4q22.1	Cytoplasm	hect domain and RLD 5	Major E3 ligase for ISG15 conjugation.
HERC6	4q22.1	Cytoplasm	hect domain and RLD 6	E3 ubiquitin-protein ligase
DDX60	4q32.3	Cytoplasm	DEAD (Asp-Glu-Ala-Asp) box polypeptide 60	ATP and RNA binding
IFITM3	11p15.5	Plasma Membrane	interferon induced transmembrane protein 3 (1-8U)	IFN-induced antiviral protein that mediates cellular innate immunity by inhibiting the early steps of replication.
SERPING1	11q12-q13.1	Extracellular Space	serpin peptidase inhibitor, clade G (C1 inhibitor), member 1, (angioedema, hereditary)	Activation of the C1 complex is under control of the C1-inhibitor.

### The association between the gene score and the pegIFN/RBV treatment response

#### Overall study population

To evaluate the association between this scoring method and the pegIFN/ribavirin treatment response, we divided the subjects into high and low gene score groups. By performing a ROC analysis, we established a cut-off value of 8 for the gene score at week 1. Patients with a high gene score (≥ 8) had significantly greater SVR rates than those with a low gene score (< 8) (83.3% vs. 20.0%, OR = 4.8, 95% C.I = 1.56–14.74, *p* = 0.017). Similarly, the patients with low gene scores had significantly higher risk of failing to achieve cEVR compared with those with high gene scores (80.0% vs. 16.7%, OR = 4.8, 95% C.I = 1.56–14.74, *p* = 0.017) (Supplementary Table S5).

**Figure 1:**
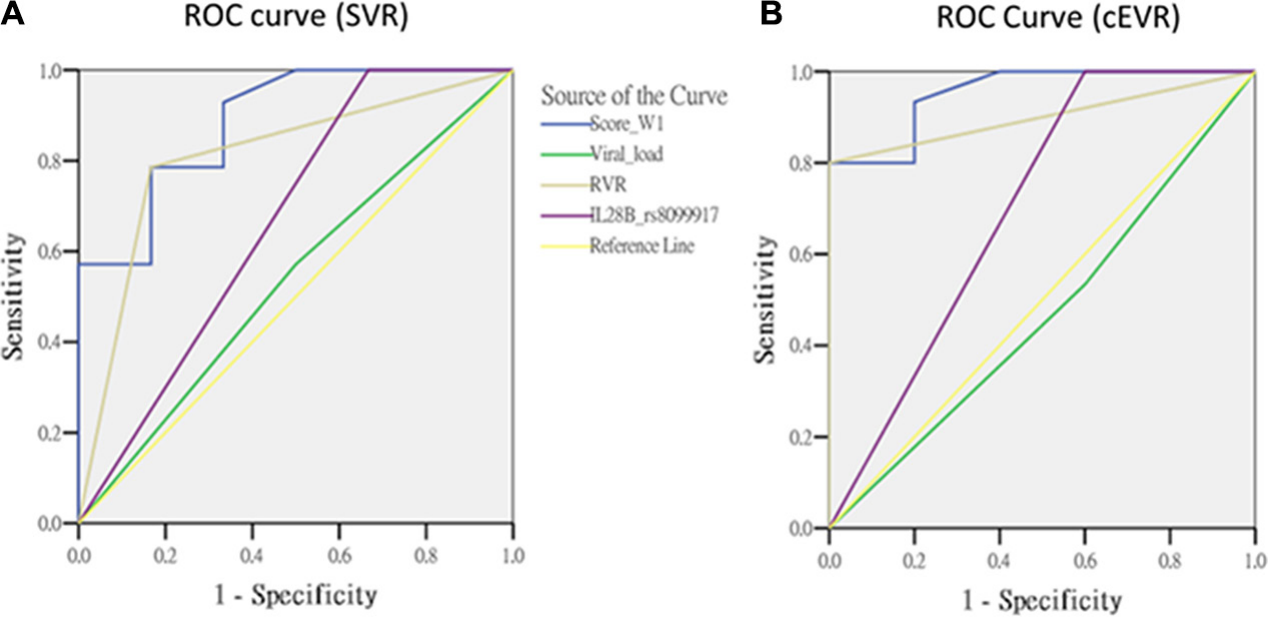
The comparisons of predictors in overall cases

The important predictors for pegIFN/ribavirin therapy treatment outcomes include the RVR, viral load and IL28B genotype. RVR is well-known as the single best predictor for SVR [19]. We compared the predictive performance among this scoring method and the traditional predictors in the overall cases by calculating the area under the ROC curve (AUC). The AUC of week 1 gene score (AUC = 0.89, *p* = 0.0074) was substantially higher than that of RVR (AUC = 0.81, *p* = 0.032) for the prediction of SVR. The AUC value of week 1 gene score for the prediction of cEVR (AUC = 0.95, *p* = 0.003) was also substantially higher than that of RVR (AUC = 0.90, *p* = 0.0088). However, both of the differences did not reach statistical significance. (RVR vs. gene score: *p* = 0.2998 for SVR; *p* = 0.4384 for cEVR). (Figure 1 and Table 3).

**Table 3:** The area under the ROC curve in overall cases

Variable	SVR				cEVR			
	AUC	95% C.I	*p*-value	Predictor vs. gene score *p*-value	AUC	95% C.I	*p*-value	Predictor vs. gene score *p*-value
Gene score (W1)	0.89	(0.73 ~ 1.04)	0.0074	Reference	0.95	(0.86 ~ 1.05)	0.0030	Reference
Viral load	0.54	(0.25 ~ 0.82)	0.8046	0.0525	0.47	(0.17 ~ 0.76)	0.8273	0.0013
RVR	0.81	(0.59 ~ 1.03)	0.0320	0.2998	0.90	(0.76 ~ 1.04)	0.0088	0.4384
IL28B rs8099917	0.67	(0.38 ~ 0.96)	0.2482	0.1072	0.70	(0.39 ~ 1.01)	0.1904	0.1032

p.s. AUC: area under the ROC curve.

### Subpopulation with IL28B TT genotypes

Because the IL28B rs8099917 TT genotype was a favorable predictor for SVR, we stratified the study population in terms of the IL28B genotypes. We analyzed the association of the week 1 gene score and treatment response among the IL28B TT subpopulation. The SVR rate was significantly elevated in the high week 1 score group compared with the low week 1 score group in patients carrying the TT genotype (92.9% vs. 25.0%, OR = 10.5, 95% C.I = 1.46–75.4, *p* = 0.019). Furthermore, the patients with a high week 1 score had a significantly greater cEVR rate compared with those with a low week 1 score among the IL28B TT subpopulation (100% vs. 25%, *p* = 0.005) (Supplementary Table S6).

**Figure 2:**
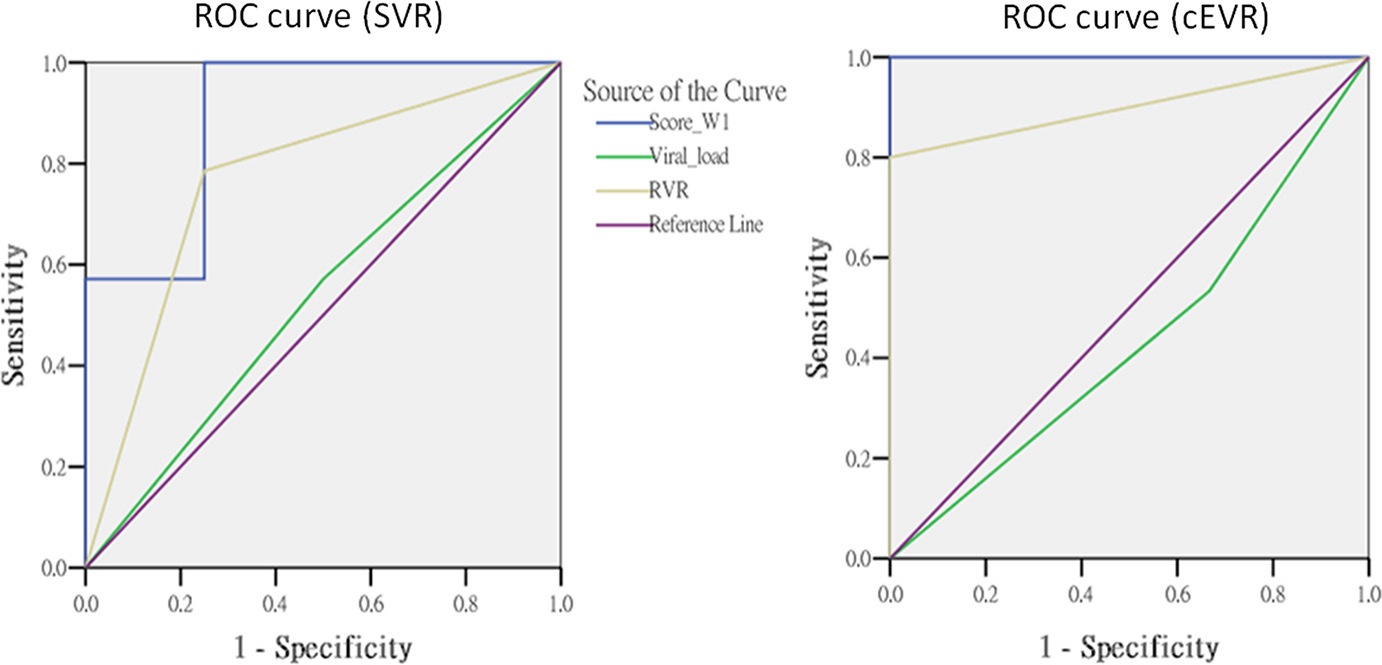
The comparisons of predictors in subjects with TT genotype

We evaluated the predictive performance between this scoring method and other predictors among the patients carrying the IL28B TT genotype. The AUC value of week 1 gene score (AUC = 0.89, *p* = 0.0195) to predict SVR was significantly higher than that of RVR (AUC = 0.77, *p* = 0.1112) (RVR vs. gene score: *p* = 0.0413). The week 1 gene score had an excellent predictive performance for cEVR (AUC = 1.00, *p* = 0.0077) that was superior to the RVR with borderline significance (AUC = 0.90, *p* = 0.0330) (RVR vs. gene score: *p* = 0.0614) (Figure 2 and Table 4).

**Table 4:** The area under the ROC curve in subjects with IL28B rs8099917 TT genotype

Variable	SVR				cEVR			
	AUC	95% C.I	*p*-value	Predictor vs. gene score *p*-value	AUC	95% C.I	*p*-value	Predictor vs. gene score *p*-value
Gene score (W1)	0.89	(0.69 ~ 1.09)	0.0195	Reference	1.00	(1.00~ 1.00)	0.0077	Reference
Viral load	0.54	(0.21 ~ 0.87)	0.8318	0.1294	0.43	(0.08 ~ 0.79)	0.7223	0.0158
RVR	0.77	(0.49 ~ 1.05)	0.1112	0.0413	0.90	(0.75 ~ 1.05)	0.0330	0.0614

p.s. AUC: area under the ROC curve.

### Model fitness analysis

We analyzed the model fitness using the Akaike information criterion (AIC), Schwarz Bayesian information criterion (BIC) and likelihood ratio test. In the overall cases, the AIC and BIC values were comparable between the week 1 gene score and RVR. The model with a single parameter (gene score or RVR) effectively predicted the SVR. In patients carrying the IL28B TT genotype, all indicators of model fitness showed that the week 1 gene score was the best predictive model for SVR (Table 5).

**Table 5:** Regression models and measurement of model fitness by AIC and BIC for SVR

Predictors	Overall cases				IL28B TT genotype	
	AIC	BIC	Likelihood ratio test *p*-value	AIC	BIC	Likelihood ratio test *p*-value
Gene score (W1)	8.597	10.868	0.008	7.653	9.434	0.007
RVR	8.522	10.793	0.002	8.241	10.021	0.049
Viral load	9.531	11.802	0.554	8.728	10.508	0.800
Viral load + W1 score	15.942	19.348	0.018	12.722	15.393	0.025
Viral load + RVR	15.251	18.657	0.007	15.443	18.115	0.130

p.s. (1) Overall cases: SVR *n* = 16, non-SVR *n* = 7; (2) Patients carrying IL28B rs8099917 TT genotype: SVR *n* = 14, non-SVR *n* = 4; (3) Model fitting information was assessed by Akaike information criterion (AIC) and the Schwarz Bayesian information criterion (BIC).

### Network analysis

**Figure 3:**
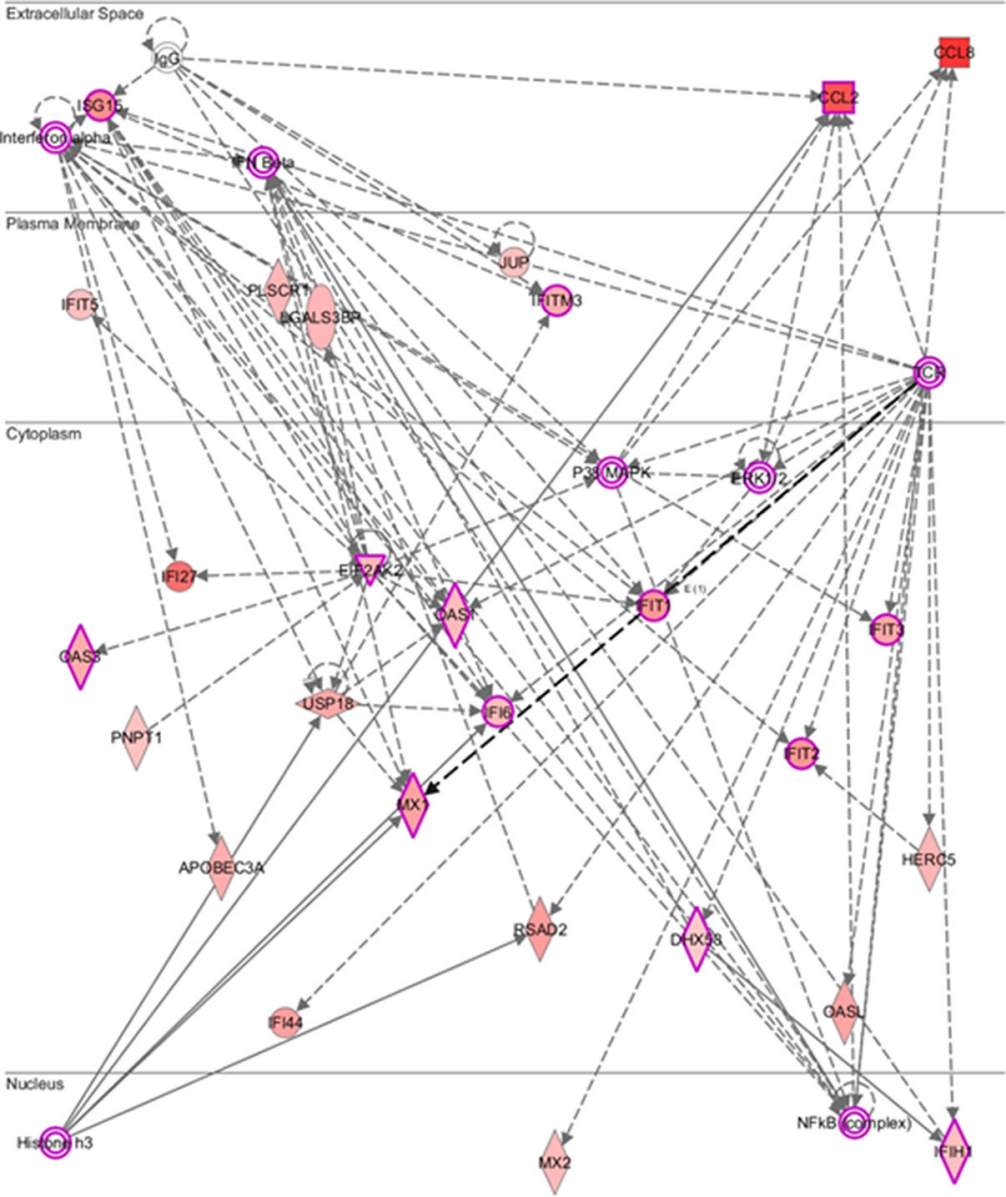
Molecular network predicted by core analysis in Ingenuity pathway analysis (IPA) p.s The scheme showed the molecular network of 43 differentially expressed genes analyzed by the ingenuity pathway analysis (IPA). The color gradient in the network indicates the strength of expression denoted by fold change. (→) indicates direct interaction between the transcript products; (—>) indicates indirect interaction between the transcript products.

Figure 3 showed the molecular network of 43 differentially expressed genes analyzed by the ingenuity pathway analysis (IPA). We explored the gene expression signature in PBMCs during pegIFN/ribavirin therapy for HCV-1. Unsurprisingly, these genes were involved in the interferon-α, interferon-β, T-cell receptor (TCR) and NF-κB signaling pathways. Notably, histone H3, which is an important epigenetic protein, played a role in this network. Histone H3 can regulate gene expression by post-translational modifications via methylation or acetylation [20].

## DISCUSSION

In this study, we analyzed the dynamic gene expression profiles of peripheral blood mononuclear cells from HCV-1 patients in response to pegIFN/ribavirin therapy. We established a molecular predictive model for the early stratification of the responders and non-responders to pegIFN/ribavirin therapy, especially among patients carrying the favorable IL28B rs8099917 TT genotype. RVR was known to be the single best predictor for SVR [19]. The performance of this genetic model was better than the traditional predictors, such as the RVR, viral load and IL28B genotype. This model advanced the predictive time for SVR by one week of pegIFN/ribavirin therapy compared with the RVR. It could help clinicians adopt appropriate strategies for HCV-1-infected patients at an earlier time point.

This scoring method reliably identified HCV-1 patients with an expected null response to pegIFN/ribavirin therapy using a cut-off value of less than 8. In the overall cases, the sensitivity, specificity, positive predictive value (PPV) and negative predictive value (NPV) for the prediction of both SVR and cEVR were 93.8%, 57.1%, 83.3% and 80.0%, respectively. In subpopulation of IL28B rs8099917 TT genotypes, the sensitivity, specificity, PPV and NPV for SVR prediction were 92.9%, 75.0%, 92.9%, and 75.0%, respectively. Among the patients carrying the IL28B TT genotype, the sensitivity, specificity, PPV and NPV for cEVR prediction were 93.3%, 100%, 100%, and 75.0%, respectively. Using the ROC curve analysis, the predictive performance of this scoring method was superior to the traditional predictors, such as the RVR, viral load and IL28B genotype.

In HCV-1 patients, a 48 week-peginterferon plus standard dose of ribavirin regimen has an SVR rate that ranges from 44%–79% [19, 21, 22]. Less than 1.6% of HCV-1 patients without cEVR have the opportunity to achieve a SVR [19, 23, 24]. Even extending the treatment duration from 48 weeks to 72 weeks, the SVR rate was only 38% for HCV-1 patients with partial EVR and 5% for those without cEVR [25]. The current treatment guidelines recommend the HCV-1 patients who fail to achieve an EVR at week 12 should stop pegIFN/ribavirin therapy [26]. This scoring method has reliable accuracy to predict SVR and cEVR, especially among patients carrying the IL28B rs8099917 TT genotype. Patients who are predicted to be non-cEVR based on the week 1 score should stop pegIFN/ribavirin therapy and wait for DAAs. Guided by this predictive model, clinicians could tailor individual treatment strategies as soon as possible. This method offers a cost-effective solution for the predicament of HCV-1 therapy and avoids the unnecessary adverse effects of the interferon-based regimen.

The pathway analysis showed that the most differentially expressed genes participated in the interferon-α, interferon-β, and NF-κB inflammatory signaling pathways. Previous studies showed that elevated interferon stimulated gene (ISG) expression in chronic HCV infected patients resulted in a poor response to peginterferon-based therapy [27, 28]. In this study, we identified constitutively expressed ISGs (e.g., RSAD2, IFI44, IFITM3…) that were overexpressed in the patients with a SVR. Understanding the gene expression signature in response to interferon could shed light on the molecular mechanism of HCV infection. Moreover, chronic HCV infection is characterized by the presence of phenotypically and functionally altered NK and T cells that fail to clear the virus [29, 30]. The pathway analysis showed some molecules (e.g., IFIT1, IFIT2, and IFIT3) that were indirectly involved in the T cell receptor (TCR)-mediated signaling pathway. Interestingly, Histone H3 was predicted to be the upstream regulator of RSAD2, MX1, and APOBEC3A. Histones can regulate DNA repair, replication and transcription [31] and are subject to a variety of posttranslational modifications, including acetylation, methylation, phosphorylation, and ubiquitylation [32]. Active genes typically carry high levels of lysine acetylation on their H3 tails [33], although the trimethylation of H3 lysine 9 (H3K9me3) and lysine 27 (H3K27me3) may repress gene expression [34]. The role of these differentially expressed genes in response to pegIFN/RBV therapy remains a mystery. However, the pathway analysis provided a clue for further functional studies.

There are several limitations in this pilot study. In this predictive model, we used the mean dCT of the non-SVR group as the reference dCT to calculate the fold change of individual gene expression. Due to the limited sample size, the mean dCT of the non-SVR group may not be sufficiently stable to serve as a standard reference. Therefore, a larger study population is needed to standardize the level of the reference dCT. Additionally, the appropriate cut-off value of the gene score to predict SVR most likely varies among different races. Another concern is that the specificity of this scoring method is not satisfactory to fit anyone. It is more suitable for patients with the favorable IL28B rs8099917 TT genotype. Because the frequency of the IL28B rs8099917 TT genotype was 83.3% in this study population, this predictive model could be applied for most HCV-1 patients. Moreover, some components of this predictive model were ISGs. The mediators that elicit the interferon signaling pathway may also interfere with the gene expression of ISGs. The interpretation of this predictive model should be approached with caution for patients co-infected with other viruses, inflammatory diseases and malignancies. This predictive model requires more studies to confirm the value of clinical application.

## MATERIALS AND METHODS

### Subjects

A total of 27 treatment naïve chronic hepatitis C patients were enrolled from Kaohsiung Medical University Hospital. The inclusion criteria were as follows: (a) adults aged more than 18 years with anti-HCV and detectable serum HCV RNA for more than 6 months; (b) infection with HCV genotype 1; and (c) serum ALT (alanine aminotransferase) increased by more than 1.5-fold over the normal range. The exclusion criteria were as follows: coinfection with hepatitis B, hepatitis D or human immunodeficiency virus and the presence of decompensated liver cirrhosis, primary biliary cirrhosis, autoimmune hepatitis, sclerosing cholangitis, α_1_-antitrypsin deficiency, Wilson disease, psychiatric conditions, current or past history of alcohol abuse (≥ 20 g daily), previous liver transplantation, or the presence of hepatocellular carcinoma or other malignancies. This study was approved by the Kaohsiung Medical University Hospital Institutional Review Board according to the guidelines of the Declaration of Helsinki and the principles of good clinical practice. Written informed consent was obtained from all participants.

### Assessment of treatment efficacy

All participants were subcutaneously treated with peginterferon α-2a (180 μg/week) plus weight-based ribavirin (1000 mg/day for weights < 75 kg and 1200 mg/day for weights ≥ 75 kg) for 48 weeks. All of the patients achieved an 80/80/80 treatment adherence of 48-week peginterferon/ribavirin, defined as receiving > 80% of peginterferon, > 80% of ribavirin and > 80% of treatment duration. A sustained virologic response (SVR) was defined as undetectable HCV RNA throughout the 24 weeks after the completion of therapy. A complete early virologic response (cEVR) was defined as undetectable HCV RNA at week 12. A partial early virologic response (pEVR) was defined as a more than 2 log_10_ IU/ml decline in the HCV RNA from baseline at week 12. A rapid virologic response (RVR) was defined as seronegativity for HCV RNA after 4 weeks of therapy. The non-SVR cohort included patients with either relapse or nonresponse.

### Cell preparation and RNA extraction

Peripheral blood was collected from the study participants at baseline and during the 1^st^ and 4^th^ weeks. Peripheral blood mononuclear cells (PBMCs) were isolated from white blood cells by the standard Ficoll-Hypaque Plus (Amersham Biosciences, Uppsala, Sweden) density gradient separation technique. RNA was purified from the PBMCs using the RiboPure™ Kit (Ambion, Applied Biosystems, Foster City, CA, USA) following the manufacturer's instructions. The RNA integrity was assessed by agarose electrophoresis. RNA samples with an A260:280 ratio > 1.8 were selected for the microarray. The isolated RNA was used as the template for one round of reverse transcription to generate cDNA with the ThermoScript RT-PCR System.

### Detection of HCV

Hepatitis C virus antibodies (anti-HCV) were detected using a third-generation commercially available enzyme-linked immunosorbent assay kit (Abbott Laboratories, Chicago, IL, USA). Serum HCV RNA was quantified using a real-time polymerase chain reaction assay [35] (RealTime HCV; Abbott Molecular, Des Plaines IL, USA; detection limit: 50 IU/ml). HCV genotypes were identified using the method proposed by Okamoto *et al*.[36].

### SNP genotyping

The IL28B *rs*8099917 genotype was significantly linked with the treatment response to PegIFN/ribavirin therapy by a genome-wide association study and replication studies in Asian cohorts [37–39]. The rs80999917 genotypes were identified using the ABI TaqMan^®^ SNP genotyping assays (Applied Biosystems, CA, USA) with the pre-designed primer and probe (ABI Assay ID: C_11710096_10) in accordance with the manufacturer›s recommendations.

### Microarray data analysis

Complementary RNA was prepared from the total RNA and hybridized to the Affymetrix Human gene 1.0 ST arrays (28869 probe sets) following the manufacturer's protocols (Affymetrix, Santa Clara, CA, USA). The hybridized arrays were scanned on an Affymetrix GeneChip^®^ scanner 3000. The initial quantification of the array images was performed by utilizing the Affymetrix GeneChip^®^ Operating Software (GCOS). Then, the data were analyzed by the R package (http://www.r-project.org) [40], which performed normalization, calculated gene expression levels, and determined the statistical significance. The threshold for significance in expression changes was set at a fold change ≥ 2 and false discovery rate (FDR) < 0.05 using the Benjamini-Hochberg procedure [41].

### Quantitative polymerase chain reaction

Differentially expressed genes obtained from the microarray data were validated by quantitative PCR. mRNA samples were used for cDNA synthesis and processed using TaqMan Gene Expression Assays (Applied Biosystems). Primer and probe sets purchased from Life Technologies were pre-designed for the respective genes (Supplementary Table S1). The reaction was executed by applying the TaqMan^®^ Gene Expression Master Mix (Applied Biosystems) on a 7500 Real-Time PCR System (Applied Biosystems). The standard thermal condition was 10 minutes at 95°C for polymerase activation, followed by 40 cycles of 95°C for 15 seconds and 60°C for 60 seconds. The expression of candidate genes was normalized to the endogenous GADPH. Relative gene expression was calculated using the ΔΔCt method.

### Bioinformatics analysis

Gene ontology (GO) analysis was performed using Protein Analysis Through Evolutionary Relationship (PANTHER, http://www.pantherdb.org) [42]. Molecular pathways and interaction networks were analyzed by Ingenuity Pathway Analysis (IPA^®^, Ingenuity Systems Inc., Redwood City, CA, USA).

### Statistical analysis

Student's *t* test was performed to analyze continuous variables. The Chi-square (*X*^2^) test or Fisher's exact test was used to assess categorical variables. The area under the curve (AUC) was calculated using receiver-operating characteristics (ROC) analysis and used to assess the capability of the predictive models. The optimum cut-off value to divide the risk strata was calculated by the Yauden index. The significance of AUC values between two predictive models was compared by the Hanley and McNeil method [43]. Model fitting analysis was evaluated by the Akaike information criterion (AIC) [44] and the Schwarz Bayesian information criterion (BIC) [45], both of which were based on the maximum likelihood estimation of the model parameters. Lower AIC and BIC values indicated a better model fit. The likelihood ratio test was used to compare the goodness-of-fit between the two models. A two-tailed *p-value* < 0.05 was considered statistically significant. All of the statistical analyses were performed using the Statistic Packages for Social Science Program (SPSS version 13.0 for windows, SPSS Inc., Chicago, IL, USA).

## CONCLUSIONS

In conclusion, this gene scoring method can reliably identify HCV-1-infected responders and non-responders to pegIFN/ribavirin therapy within one week. The performance of this predictive model was superior to traditional predictors, such as the RVR, viral load and IL28B rs8099917 genotype. The model will help clinicians adopt an appropriate strategy for chronic HCV-1 patients at an early time point.

## SUPPLEMENTARY MATERIALS FIGURES AND TABLES




