# Supplementary material for: Dynamics of PBMC gene expression in hepatitis C virus genotype 1-infected patients during combined peginterferon/ribavirin therapy
Source: Oncotarget. 2016 Aug 17;7(38):61325–35. doi: 10.18632/oncotarget.11348 (PMC5308654; doi:10.18632/oncotarget.11348)
Supplement: Supplementary file 1 [file oncotarget-07-61325-s001.pdf]

Dynamics of PBMC gene expression in hepatitis C virus genotype 1-infected patients during combined peginterferon/ribavirin therapy

Supplementary Materials

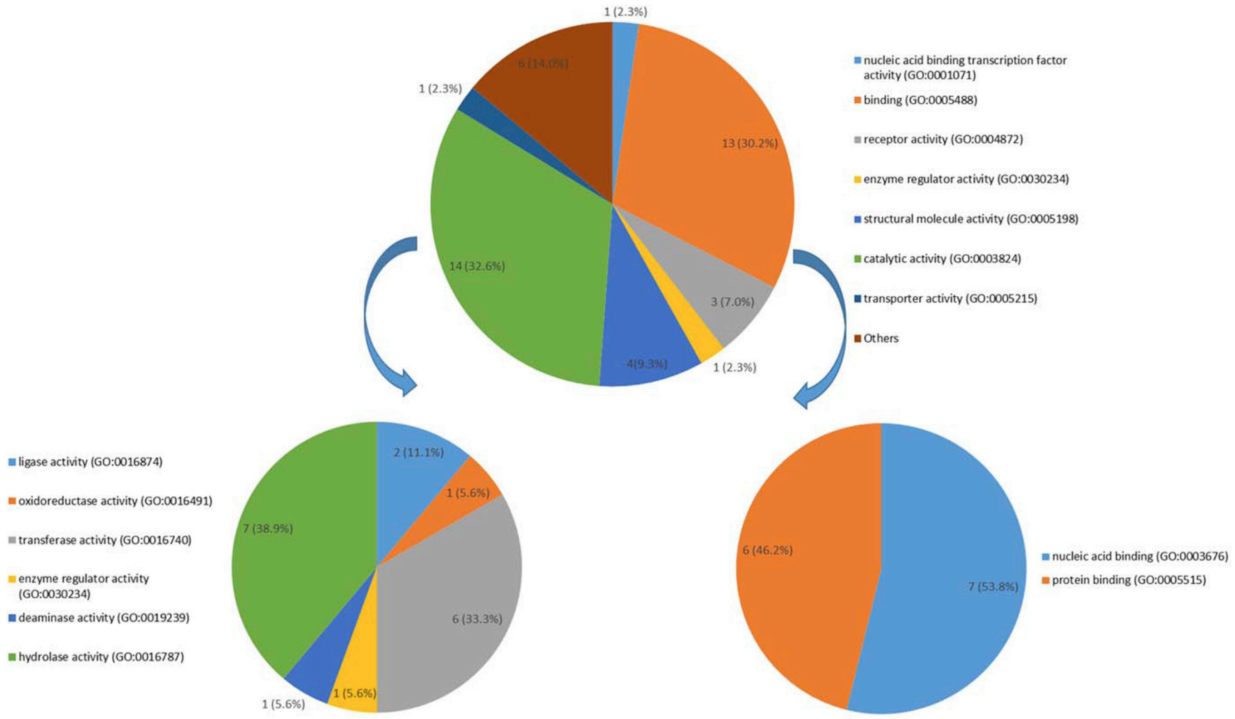

Supplementary Figure S1: Functional classification of 43 differentially expressed genes.

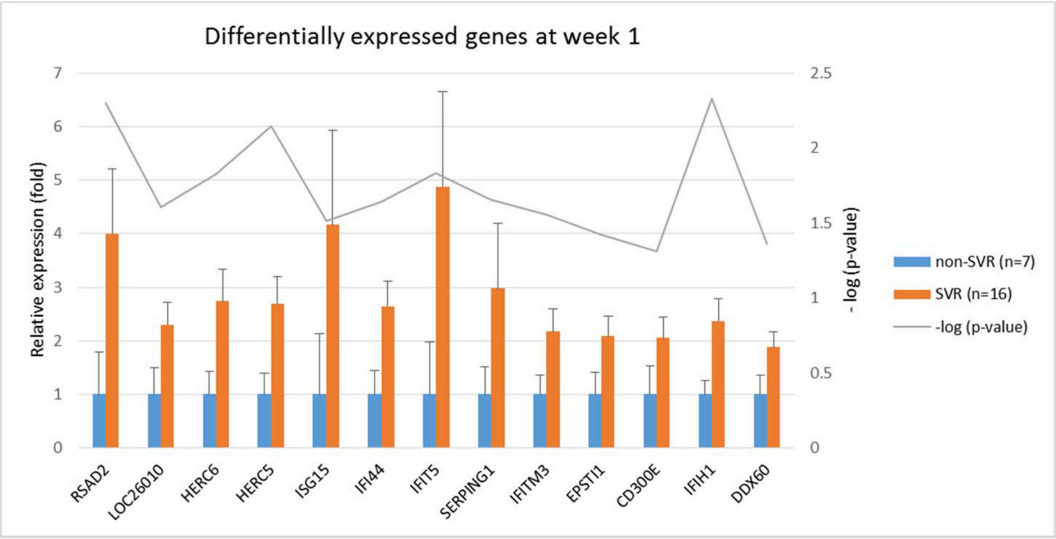

Supplementary Figure S2: Differentially expressed genes between SVR and non-SVR at week 1. p.s. The genes validated by real-time PCR with *p*-value < 0.05 were listed. The bar presents mean of fold change ± SE. The relative expression of fold change was normalized by endogenous GADPH.

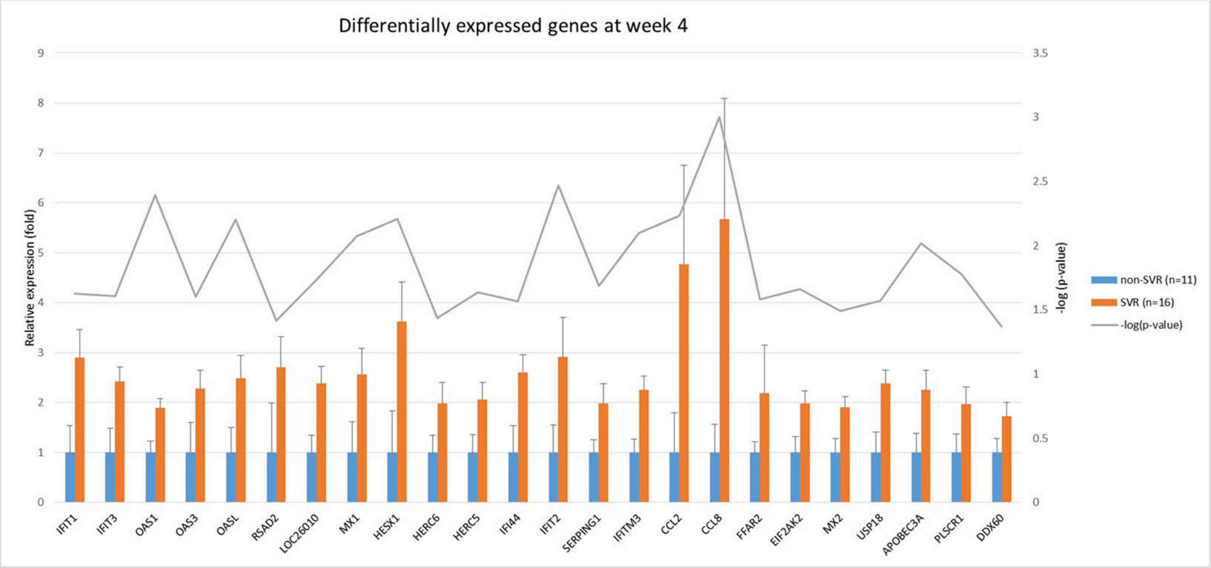

Supplementary Figure S3: Differentially expressed genes between SVR and non-SVR at week 4. p.s. The genes validated by real-time PCR with *p*-value < 0.05 were listed. The bar presents mean of fold change $\pm$ SE. The relative expression of fold change was normalized by endogenous GADPH.

Supplementary Table S1: List of 43 differentially expressed genes identified by microarray. See Supplementary_Table_S1

Supplementary Table S2: Comparison of the expression of genes between SVR and non-SVR. See Supplementary_Table_S2

Supplementary Table S3: Comparison of the expression of genes between cEVR and non-cEVR. See Supplementary_Table_S3

Supplementary Table S4: Comparison of the expression of genes between RVR and non-RVR. See Supplementary_Table_S4

Supplementary Table S5: The association between gene score and pegIFN/ribavirin treatment response in overall cases

Score (W1)	SVR	non-SVR	Fisher's *p*-value	OR (95% C.I)	cEVR	non-cEVR	Fisher's *p*-value	OR (95% C.I)
≥ 8	15 (83.3%)	3 (16.7%)	0.017	4.8 (1.56~14.74)	15 (83.3%)	3 (16.7%)	0.017	4.8 (1.56~14.74)
< 8	1 (20.0%)	4 (80.0%)			1 (20.0%)	4 (80.0%)		

p.s. gene score = the sum of fold change (RSAD2 + LOC26010 + HERC5 + HERC6 + IFI44 + SERPING1 + IFITM3 + DDX60).

Supplementary Table S6: The association between gene score and pegIFN/ribavirin treatment response in IL28B rs80999917 TT genotype

Score (W1)	SVR	non-SVR	Fisher's *p*-value	OR (95% C.I)	cEVR	non-cEVR	Fisher's *p*-value	OR (95% C.I)
≥ 8	13 (92.9%)	1 (7.1%)	0.019	10.5 (1.46~75.4)	14 (100.0%)	0 (0.0%)	0.005	~
< 8	1 (25.0%)	3 (75.0%)			1 (25.0%)	3 (75.0%)		

p.s. gene score = the sum of fold change (RSAD2 + LOC26010 + HERC5 + HERC6 + IFI44 + SERPING1 + IFITM3 + DDX60).
