# Supplementary material for: Dynamics of PBMC gene expression in hepatitis C virus genotype 1-infected patients during combined peginterferon/ribavirin therapy
Source: Oncotarget. 2016 Aug 17;7(38):61325–35. doi: 10.18632/oncotarget.11348 (PMC5308654; doi:10.18632/oncotarget.11348)
Supplement: Supplementary file 2 [file oncotarget-07-61325-s002.docx]

| Supplementary table 1. List of 43 differentially expressed genes identified by microarray | | | | | | |  |  |
| --- | --- | --- | --- | --- | --- | --- | --- | --- |
| **No** | **SYMBOL** | **HS** | **AffyID** | **ACC** | **CYTOBAND** | **Location** | **Type(s)** | **DESCRIPTION** |
| 1 | IFI27 | Hs00271467_m1 | 7976443 | NM_005532 | 14q32 | Cytoplasm | other | interferon, alpha-inducible protein 27 |
| 2 | IFI6 | Hs00242571_m1 | 7914127 | NM_022872 | 1p35 | Cytoplasm | other | interferon, alpha-inducible protein 6 |
| 3 | IFIT1 | Hs01675197_m1 | 7929065 | NM_001548 | 10q25-q26 | Cytoplasm | other | interferon-induced protein with tetratricopeptide repeats 1 |
| 4 | IFIT3 | Hs00382744_m1 | 7929052 | NM_001031683 | 10q24 | Cytoplasm | other | interferon-induced protein with tetratricopeptide repeats 3 |
| 5 | OAS1 | Hs00973637_m1 | 7958884 | NM_016816 | 12q24.1 | Cytoplasm | enzyme | 2',5'-oligoadenylate synthetase 1, 40/46kDa |
| 6 | OAS3 | Hs00196324_m1 | 7958895 | NM_006187 | 12q24.2 | Cytoplasm | enzyme | 2'-5'-oligoadenylate synthetase 3, 100kDa |
| 7 | OASL | Hs00984390_m1 | 7967117 | NM_003733 | 12q24.2 | Cytoplasm | enzyme | 2'-5'-oligoadenylate synthetase-like |
| 8 | DHX58 | Hs00225561_m1 | 8015511 | NM_024119 | 17q21.2 | Cytoplasm | enzyme | DEXH (Asp-Glu-X-His) box polypeptide 58 |
| 9 | RSAD2 | Hs00369813_m1 | 8040080 | NM_080657 | 2p25.2 | Cytoplasm | enzyme | radical S-adenosyl methionine domain containing 2 |
| 10 | LOC26010 | Hs00274702_m1 | 8047272 | AF193059 | 2q33.1 | Nucleus |  | viral DNA polymerase-transactivated protein 6 |
| 11 | SIGLEC1 | Hs00988063_m1 | 8064716 | NM_023068 | 20p13 | Plasma Membrane | other | sialic acid binding Ig-like lectin 1, sialoadhesin |
| 12 | MX1 | Hs00182073_m1 | 8068713 | NM_002462 | 21q22.3 | Cytoplasm | enzyme | myxovirus (influenza virus) resistance 1, interferon-inducible protein p78 (mouse) |
| 13 | HESX1 | Hs00172696_m1 | 8088285 | NM_003865 | 3p21.2-p21.1 | Nucleus | transcription regulator | HESX homeobox 1 |
| 14 | LAMP3 | Hs00180880_m1 | 8092348 | NM_014398 | 3q26.3-q27 | Plasma Membrane | other | lysosomal-associated membrane protein 3 |
| 15 | HERC6 | Hs00215555_m1 | 8096335 | NM_017912 | 4q22.1 | Cytoplasm | other | hect domain and RLD 6 |
| 16 | HERC5 | Hs00180943_m1 | 8096361 | NM_016323 | 4q22.1 | Cytoplasm | enzyme | hect domain and RLD 5 |
| 17 | ISG15 | Hs00192713_m1 | 7896817 | NM_005101 | 1p36.33 | Extracellular Space | other | ISG15 ubiquitin-like modifier |
| 18 | IFI44 | Hs00197427_m1 | 7902553 | NM_006417 | 1p31.1 | Cytoplasm | other | interferon-induced protein 44 |
| 19 | IFIT2 | Hs00533665_m1 | 7929047 | NM_001547 | 10q23-q25 | Cytoplasm | other | interferon-induced protein with tetratricopeptide repeats 2 |
| 20 | IFIT5 | Hs00202721_m1 | 7929072 | NM_012420 | 10q23.31 | Plasma Membrane | other | interferon-induced protein with tetratricopeptide repeats 5 |
| 21 | SERPING1 | Hs00934330_m1 | 7940028 | NM_000062 | 11q12-q13.1 | Extracellular Space | other | serpin peptidase inhibitor, clade G (C1 inhibitor), member 1, (angioedema, hereditary) |
| 22 | IFITM3 | Hs03057129_s1 | 7945371 | NM_021034 | 11p15.5 | Plasma Membrane | other | interferon induced transmembrane protein 3 (1-8U) |
| 23 | EPSTI1 | Hs01566789_m1 | 7971296 | NM_001002264 | 13q13.3 | Other | other | epithelial stromal interaction 1 (breast) |
| 24 | CCL2 | Hs00234140_m1 | 8006433 | NM_002982 | 17q11.2-q12 | Extracellular Space | cytokine | chemokine (C-C motif) ligand 2 |
| 25 | CCL8 | Hs00271615_m1 | 8006453 | NM_005623 | 17q11.2 | Extracellular Space | cytokine | chemokine (C-C motif) ligand 8 |
| 26 | JUP | Hs00158408_m1 | 8015412 | NM_002230 | 17q21 | Plasma Membrane | other | junction plakoglobin |
| 27 | CD300E | Hs00417308_m1 | 8018189 | NM_181449 | 17q25.1 | Plasma Membrane | other | CD300e molecule |
| 28 | LGALS3BP | Hs01003086_m1 | 8018975 | NM_005567 | 17q25 | Plasma Membrane | transmembrane receptor | lectin, galactoside-binding, soluble, 3 binding protein |
| 29 | FFAR2 | Hs00271142_s1 | 8027862 | NM_005306 | 19q13.1 | Plasma Membrane | G-protein coupled receptor | free fatty acid receptor 2 |
| 30 | EIF2AK2 | Hs00169345_m1 | 8051501 | NM_002759 | 2p22-p21 | Cytoplasm | kinase | eukaryotic translation initiation factor 2-alpha kinase 2 |
| 31 | PNPT1 | Hs00396733_g1 | 8052331 | NM_033109 | 2p15 | Cytoplasm | enzyme | polyribonucleotide nucleotidyltransferase 1 |
| 32 | IFIH1 | Hs01070332_m1 | 8056285 | NM_022168 | 2q24 | Nucleus | enzyme | interferon induced with helicase C domain 1 |
| 33 | PGAP1 | Hs01088726_m1 | 8057959 | NM_024989 | 2q33.1 | Cytoplasm | enzyme | GPI deacylase |
| 34 | MX2 | Hs01550808_m1 | 8068697 | NM_002463 | 21q22.3 | Nucleus | enzyme | myxovirus (influenza virus) resistance 2 (mouse) |
| 35 | USP18 | Hs00276441_m1 | 8071155 | NM_017414 | 22q11.21 | Cytoplasm | peptidase | ubiquitin specific peptidase 18 |
| 36 | APOBEC3A | Hs00377444_m1 | 8073056 | NM_145699 | 22q13.1-q13.2 | Cytoplasm | enzyme | apolipoprotein B mRNA editing enzyme, catalytic polypeptide-like 3A |
| 37 | PLSCR1 | Hs00275514_m1 | 8091327 | NM_021105 | 3q23 | Plasma Membrane | enzyme | phospholipid scramblase 1 |
| 38 | DDX60 | Hs00214153_m1 | #N/A | #N/A | 4q32.3 | Cytoplasm | enzyme | DEAD (Asp-Glu-Ala-Asp) box polypeptide 60 |
| 39 | CTSL1 | Hs00377632_m1 | 8156228 | NM_001912 | 9q21.33 | Cytoplasm | peptidase | cathepsin L1 |
| 40 | PPBP | Hs00234077_m1 | #N/A | #N/A | 4q12-q13 | Extracellular Space | cytokine | pro-platelet basic protein (chemokine (C-X-C motif) ligand 7) |
| 41 | GNG11 | Hs00914578_m1 | #N/A | #N/A | 7q21 | Plasma Membrane | enzyme | guanine nucleotide binding protein (G protein), gamma 11 |
| 42 | ACAA1 | Hs01576070_m1 | 8086216 | AK127051 | 3p23-p22 | Cytoplasm | enzyme | acetyl-Coenzyme A acyltransferase 1 (peroxisomal 3-oxoacyl-Coenzyme A thiolase) |
| 43 | FCER1A | Hs00758600_m1 | #N/A | #N/A | 1q23 | Plasma Membrane | transmembrane receptor | Fc fragment of IgE, high affinity I, receptor for; alpha polypeptide |

| Supplementary table 2. Comparison of the expression of genes between SVR and non-SVR | | | | | | | | | |  |  |  |  |  |  |  |  |  |  |  |  |  |  |  |  |  |  |  |
| --- | --- | --- | --- | --- | --- | --- | --- | --- | --- | --- | --- | --- | --- | --- | --- | --- | --- | --- | --- | --- | --- | --- | --- | --- | --- | --- | --- | --- |
|  |  |  | Week 0 |  |  |  |  |  |  |  |  | Week 1 |  |  |  |  |  |  |  |  | Week 4 |  |  |  |  |  |  |  |
|  | dCT |  | SVR (n=16) | |  | non-SVR (n=11) | |  |  |  |  | SVR (n=16) | |  | non-SVR (n=7) | |  |  |  |  | SVR (n=16) | |  | non-SVR (n=11) | |  |  |  |
|  | Gene | Accession no. | Mean | SD |  | Mean | SD | ddCt | Fold | p-value |  | Mean | SD |  | Mean | SD | ddCt | Fold | p-value |  | Mean | SD |  | Mean | SD | ddCt | Fold | p-value |
| G01 | IFI27 | Hs00271467_m1 | 6.61 | 3.35 |  | 7.81 | 3.22 | -1.20 | 2.30 | 0.3618 |  | 2.09 | 2.56 |  | 3.63 | 3.72 | -1.55 | 2.93 | 0.2577 |  | 0.52 | 1.31 |  | 2.50 | 3.98 | -1.98 | 3.95 | 0.1382 |
| G02 | IFI6 | Hs00242571_m1 | 4.51 | 2.25 |  | 5.48 | 2.20 | -0.97 | 1.96 | 0.2768 |  | 2.52 | 1.60 |  | 4.29 | 2.55 | -1.77 | 3.42 | 0.0543 |  | 2.04 | 1.56 |  | 2.96 | 1.99 | -0.92 | 1.90 | 0.1881 |
| G03 | IFIT1 | Hs01675197_m1 | 8.10 | 1.97 |  | 8.93 | 1.55 | -0.83 | 1.77 | 0.2567 |  | 6.61 | 1.61 |  | 8.11 | 2.15 | -1.50 | 2.83 | 0.0777 |  | 5.81 | 0.91 |  | 7.35 | 1.85 | -1.53 | 2.90 | 0.0237 |
| G04 | IFIT3 | Hs00382744_m1 | 4.65 | 1.64 |  | 5.34 | 1.26 | -0.69 | 1.61 | 0.2526 |  | 3.77 | 1.29 |  | 4.94 | 1.71 | -1.17 | 2.25 | 0.0849 |  | 3.01 | 0.63 |  | 4.29 | 1.58 | -1.28 | 2.43 | 0.0248 |
| G05 | OAS1 | Hs00973637_m1 | 1.74 | 1.37 |  | 2.24 | 1.18 | -0.50 | 1.41 | 0.3357 |  | 1.54 | 0.83 |  | 2.16 | 0.90 | -0.63 | 1.55 | 0.1173 |  | 0.89 | 0.55 |  | 1.81 | 0.96 | -0.92 | 1.89 | 0.0041 |
| G06 | OAS3 | Hs00196324_m1 | 3.24 | 1.64 |  | 3.65 | 1.30 | -0.41 | 1.33 | 0.4924 |  | 2.86 | 1.15 |  | 3.75 | 1.88 | -0.88 | 1.85 | 0.1776 |  | 2.22 | 0.91 |  | 3.41 | 1.68 | -1.19 | 2.28 | 0.0251 |
| G07 | OASL | Hs00984390_m1 | 3.00 | 1.50 |  | 3.78 | 1.08 | -0.78 | 1.72 | 0.1503 |  | 2.98 | 1.09 |  | 3.64 | 1.26 | -0.66 | 1.58 | 0.2185 |  | 2.03 | 0.88 |  | 3.34 | 1.41 | -1.31 | 2.48 | 0.0063 |
| G08 | DHX58 | Hs00225561_m1 | 7.19 | 0.99 |  | 7.11 | 0.95 | 0.08 | 0.95 | 0.8433 |  | 5.83 | 0.98 |  | 6.54 | 1.22 | -0.71 | 1.64 | 0.1509 |  | 5.61 | 0.72 |  | 6.17 | 1.01 | -0.56 | 1.47 | 0.1054 |
| G09 | RSAD2 | Hs00369813_m1 | 4.30 | 2.03 |  | 5.10 | 1.65 | -0.80 | 1.74 | 0.2926 |  | 1.95 | 1.29 |  | 3.95 | 1.67 | -2.00 | 4.00 | 0.005 |  | 1.45 | 0.95 |  | 2.89 | 1.92 | -1.44 | 2.71 | 0.0383 |
| G10 | LOC26010 | Hs00274702_m1 | 7.15 | 1.44 |  | 7.83 | 1.27 | -0.68 | 1.61 | 0.2143 |  | 6.21 | 0.98 |  | 7.42 | 1.35 | -1.20 | 2.30 | 0.0248 |  | 5.53 | 0.80 |  | 6.78 | 1.41 | -1.25 | 2.38 | 0.0183 |
| G11 | SIGLEC1 | Hs00988063_m1 | 3.63 | 2.80 |  | 5.00 | 2.80 | -1.37 | 2.58 | 0.225 |  | 2.55 | 1.92 |  | 4.07 | 2.77 | -1.52 | 2.86 | 0.2222 |  | 1.78 | 1.71 |  | 2.82 | 2.18 | -1.04 | 2.06 | 0.1755 |
| G12 | MX1 | Hs00182073_m1 | 0.15 | 1.85 |  | 0.96 | 1.74 | -0.81 | 1.75 | 0.2621 |  | -0.13 | 1.19 |  | 0.85 | 1.47 | -0.98 | 1.98 | 0.1046 |  | -1.07 | 1.00 |  | 0.30 | 1.47 | -1.36 | 2.57 | 0.0084 |
| G13 | HESX1 | Hs00172696_m1 | 10.36 | 1.84 |  | 11.56 | 1.74 | -1.20 | 2.29 | 0.1025 |  | 9.63 | 1.71 |  | 10.71 | 1.70 | -1.08 | 2.12 | 0.2005 |  | 8.47 | 1.21 |  | 10.32 | 2.02 | -1.85 | 3.62 | 0.0062 |
| G14 | LAMP3 | Hs00180880_m1 | 9.00 | 1.46 |  | 8.88 | 1.61 | 0.12 | 0.92 | 0.8401 |  | 6.73 | 1.51 |  | 7.75 | 1.45 | -1.02 | 2.02 | 0.1469 |  | 6.27 | 1.22 |  | 7.06 | 1.64 | -0.79 | 1.73 | 0.1621 |
| G15 | HERC6 | Hs00215555_m1 | 4.67 | 1.27 |  | 5.33 | 1.31 | -0.67 | 1.59 | 0.1976 |  | 2.75 | 1.13 |  | 4.20 | 1.38 | -1.45 | 2.74 | 0.0148 |  | 2.43 | 0.96 |  | 3.41 | 1.37 | -0.99 | 1.98 | 0.0367 |
| G16 | HERC5 | Hs00180943_m1 | 3.82 | 1.44 |  | 4.43 | 1.21 | -0.61 | 1.53 | 0.2595 |  | 2.82 | 1.00 |  | 4.25 | 1.20 | -1.43 | 2.69 | 0.0072 |  | 2.39 | 0.88 |  | 3.43 | 1.36 | -1.04 | 2.06 | 0.0231 |
| G17 | ISG15 | Hs00192713_m1 | 4.96 | 2.66 |  | 6.60 | 2.52 | -1.64 | 3.11 | 0.1207 |  | 4.94 | 1.74 |  | 7.00 | 2.42 | -2.06 | 4.16 | 0.0304 |  | 4.01 | 1.90 |  | 5.65 | 2.28 | -1.64 | 3.12 | 0.0525 |
| G18 | IFI44 | Hs00197427_m1 | 2.07 | 1.29 |  | 2.90 | 1.88 | -0.83 | 1.78 | 0.2207 |  | 1.47 | 1.07 |  | 2.87 | 1.64 | -1.40 | 2.64 | 0.0226 |  | 0.83 | 0.73 |  | 2.21 | 1.73 | -1.38 | 2.61 | 0.0272 |
| G19 | IFIT2 | Hs00533665_m1 | 2.22 | 1.56 |  | 3.06 | 1.01 | -0.84 | 1.78 | 0.1325 |  | 2.76 | 1.22 |  | 3.79 | 1.45 | -1.03 | 2.05 | 0.0929 |  | 1.77 | 1.11 |  | 3.32 | 1.35 | -1.54 | 2.91 | 0.0034 |
| G20 | IFIT5 | Hs00202721_m1 | 6.03 | 1.91 |  | 6.80 | 1.70 | -0.77 | 1.71 | 0.2926 |  | 4.53 | 1.64 |  | 6.82 | 2.41 | -2.29 | 4.88 | 0.0146 |  | 4.58 | 2.35 |  | 5.40 | 2.55 | -0.82 | 1.76 | 0.3978 |
| G21 | SERPING1 | Hs00934330_m1 | 4.48 | 1.61 |  | 5.63 | 1.32 | -1.15 | 2.22 | 0.0614 |  | 3.37 | 1.33 |  | 4.94 | 1.59 | -1.57 | 2.98 | 0.0223 |  | 3.10 | 0.95 |  | 4.09 | 1.11 | -0.99 | 1.98 | 0.0205 |
| G22 | IFITM3 | Hs03057129_s1 | -2.10 | 1.57 |  | -1.08 | 1.16 | -1.01 | 2.02 | 0.0804 |  | -3.27 | 1.00 |  | -2.15 | 1.13 | -1.12 | 2.17 | 0.0276 |  | -3.73 | 0.82 |  | -2.56 | 1.29 | -1.17 | 2.25 | 0.008 |
| G23 | EPSTI1 | Hs01566789_m1 | 0.36 | 1.71 |  | 1.18 | 1.63 | -0.82 | 1.76 | 0.2259 |  | -0.03 | 0.97 |  | 1.03 | 1.25 | -1.06 | 2.09 | 0.0379 |  | -0.54 | 0.97 |  | 0.32 | 1.21 | -0.87 | 1.82 | 0.0496 |
| G24 | CCL2 | Hs00234140_m1 | 6.56 | 2.29 |  | 7.66 | 2.18 | -1.11 | 2.15 | 0.2197 |  | 5.99 | 2.13 |  | 7.80 | 2.47 | -1.81 | 3.50 | 0.0882 |  | 4.54 | 1.77 |  | 6.79 | 2.11 | -2.26 | 4.77 | 0.0058 |
| G25 | CCL8 | Hs00271615_m1 | 7.61 | 2.14 |  | 9.16 | 2.06 | -1.55 | 2.92 | 0.0729 |  | 8.54 | 2.58 |  | 9.89 | 1.60 | -1.36 | 2.56 | 0.2158 |  | 6.95 | 1.86 |  | 9.45 | 1.47 | -2.50 | 5.67 | 0.001 |
| G26 | JUP | Hs00158408_m1 | 4.45 | 2.27 |  | 4.99 | 1.93 | -0.54 | 1.46 | 0.5234 |  | 2.70 | 1.83 |  | 3.99 | 2.41 | -1.29 | 2.44 | 0.1724 |  | 2.31 | 1.64 |  | 3.11 | 1.74 | -0.80 | 1.74 | 0.2363 |
| G27 | CD300E | Hs00417308_m1 | 2.07 | 0.81 |  | 2.25 | 0.73 | -0.18 | 1.13 | 0.5604 |  | 1.15 | 0.91 |  | 2.19 | 1.45 | -1.04 | 2.05 | 0.0486 |  | 1.24 | 1.08 |  | 1.69 | 1.08 | -0.46 | 1.37 | 0.2879 |
| G28 | LGALS3BP | Hs01003086_m1 | 4.25 | 2.60 |  | 5.22 | 2.89 | -0.97 | 1.96 | 0.3704 |  | 3.06 | 1.96 |  | 4.85 | 2.94 | -1.79 | 3.47 | 0.0974 |  | 2.35 | 2.24 |  | 3.36 | 2.06 | -1.01 | 2.01 | 0.2461 |
| G29 | FFAR2 | Hs00271142_s1 | 5.53 | 1.80 |  | 6.43 | 1.38 | -0.90 | 1.87 | 0.1747 |  | 5.97 | 1.84 |  | 6.04 | 0.92 | -0.07 | 1.05 | 0.9246 |  | 4.96 | 1.33 |  | 6.09 | 1.04 | -1.13 | 2.19 | 0.0263 |
| G30 | EIF2AK2 | Hs00169345_m1 | 2.52 | 1.19 |  | 3.15 | 1.11 | -0.63 | 1.54 | 0.1787 |  | 2.20 | 0.95 |  | 2.97 | 0.92 | -0.77 | 1.71 | 0.085 |  | 1.50 | 0.60 |  | 2.49 | 1.17 | -0.99 | 1.99 | 0.0218 |
| G31 | PNPT1 | Hs00396733_g1 | 4.86 | 0.84 |  | 5.46 | 1.12 | -0.60 | 1.52 | 0.1223 |  | 5.60 | 0.92 |  | 6.23 | 0.68 | -0.63 | 1.55 | 0.1204 |  | 4.94 | 0.88 |  | 5.68 | 1.02 | -0.75 | 1.68 | 0.0538 |
| G32 | IFIH1 | Hs01070332_m1 | 5.15 | 0.98 |  | 5.63 | 0.95 | -0.48 | 1.39 | 0.219 |  | 3.79 | 0.88 |  | 5.04 | 0.85 | -1.25 | 2.37 | 0.0047 |  | 3.55 | 0.85 |  | 4.37 | 1.07 | -0.82 | 1.77 | 0.035 |
| G33 | PGAP1 | Hs01088726_m1 | 10.01 | 0.95 |  | 10.46 | 1.03 | -0.45 | 1.37 | 0.2529 |  | 9.15 | 1.01 |  | 9.98 | 0.84 | -0.83 | 1.77 | 0.0719 |  | 8.94 | 0.96 |  | 9.21 | 0.80 | -0.26 | 1.20 | 0.4605 |
| G34 | MX2 | Hs01550808_m1 | 0.66 | 1.41 |  | 1.18 | 1.33 | -0.52 | 1.43 | 0.3491 |  | -0.15 | 1.03 |  | 0.77 | 1.12 | -0.92 | 1.89 | 0.0669 |  | -0.64 | 0.65 |  | 0.29 | 1.19 | -0.93 | 1.91 | 0.0323 |
| G35 | USP18 | Hs00276441_m1 | 5.37 | 1.76 |  | 5.63 | 1.65 | -0.25 | 1.19 | 0.7083 |  | 4.26 | 1.33 |  | 5.14 | 1.45 | -0.88 | 1.84 | 0.1696 |  | 3.23 | 0.60 |  | 4.49 | 1.57 | -1.25 | 2.38 | 0.0267 |
| G36 | APOBEC3A | Hs00377444_m1 | 2.82 | 1.59 |  | 3.78 | 1.51 | -0.97 | 1.95 | 0.1269 |  | 2.74 | 1.38 |  | 3.55 | 1.20 | -0.81 | 1.76 | 0.19 |  | 1.84 | 0.94 |  | 3.01 | 1.23 | -1.17 | 2.25 | 0.0096 |
| G37 | PLSCR1 | Hs00275514_m1 | 0.87 | 1.15 |  | 1.72 | 0.94 | -0.84 | 1.80 | 0.0554 |  | 1.07 | 0.85 |  | 1.84 | 0.99 | -0.77 | 1.71 | 0.0687 |  | 0.48 | 0.87 |  | 1.47 | 1.12 | -0.98 | 1.98 | 0.0168 |
| G38 | DDX60 | Hs00214153_m1 | 3.23 | 1.29 |  | 3.61 | 0.98 | -0.39 | 1.31 | 0.4095 |  | 2.23 | 0.85 |  | 3.14 | 1.13 | -0.91 | 1.88 | 0.0438 |  | 1.72 | 0.80 |  | 2.51 | 1.11 | -0.79 | 1.73 | 0.0425 |
| G39 | CTSL1 | Hs00377632_m1 | 3.32 | 2.97 |  | 3.32 | 0.94 | 0.00 | 1.00 | 0.9963 |  | 2.45 | 0.74 |  | 3.19 | 1.19 | -0.74 | 1.67 | 0.0817 |  | 2.22 | 0.82 |  | 2.63 | 0.88 | -0.40 | 1.32 | 0.2343 |
| G40 | PPBP | Hs00234077_m1 | 0.64 | 2.69 |  | 1.61 | 2.19 | -0.97 | 1.96 | 0.3315 |  | 1.07 | 1.39 |  | 1.62 | 1.55 | -0.55 | 1.47 | 0.4069 |  | 1.88 | 1.99 |  | 1.44 | 1.51 | 0.44 | 0.74 | 0.5409 |
| G41 | GNG11 | Hs00914578_m1 | 5.16 | 2.66 |  | 5.86 | 2.32 | -0.71 | 1.63 | 0.4832 |  | 5.07 | 1.24 |  | 5.91 | 1.57 | -0.84 | 1.79 | 0.1807 |  | 5.29 | 1.43 |  | 5.20 | 1.49 | 0.08 | 0.94 | 0.8854 |
| G42 | ACAA1 | Hs01576070_m1 | 4.13 | 2.96 |  | 3.93 | 0.55 | 0.20 | 0.87 | 0.8316 |  | 4.47 | 0.46 |  | 4.62 | 0.49 | -0.15 | 1.11 | 0.4979 |  | 4.36 | 0.77 |  | 4.42 | 0.37 | -0.06 | 1.05 | 0.7977 |
| G43 | FCER1A | Hs00758600_m1 | 2.80 | 2.73 |  | 3.02 | 0.93 | -0.23 | 1.17 | 0.7952 |  | 3.99 | 0.81 |  | 4.94 | 1.75 | -0.95 | 1.93 | 0.0858 |  | 4.46 | 1.61 |  | 3.80 | 0.80 | 0.66 | 0.63 | 0.2202 |
| Endogenous control: GADPH | | |  |  |  |  |  |  |  |  |  |  |  |  |  |  |  |  |  |  |  |  |  |  |  |  |  |  |

| Supplementary table 3. Comparison of the expression of genes between cEVR and non-cEVR | | | | | | | | | |  |  |  |  |  |  |  |  |  |  |  |  |  |  |  |  |  |  |  |
| --- | --- | --- | --- | --- | --- | --- | --- | --- | --- | --- | --- | --- | --- | --- | --- | --- | --- | --- | --- | --- | --- | --- | --- | --- | --- | --- | --- | --- |
|  |  |  | Week 0 |  |  |  |  |  |  |  |  | Week 1 |  |  |  |  |  |  |  |  | Week 4 |  |  |  |  |  |  |  |
|  | dCT |  | cEVR (n=16) | |  | non-cEVR (n=11) | | |  |  |  | cEVR (n=16) | |  | non-cEVR (n=7) | |  |  |  |  | cEVR (n=16) | |  | non-cEVR (n=11) | | |  |  |
|  | Gene | Accession no. | Mean | SD |  | Mean | SD | ddCt | Fold | p-value |  | Mean | SD |  | Mean | SD | ddCt | Fold | p-value |  | Mean | SD |  | Mean | SD | ddCt | Fold | p-value |
| G01 | IFI27 | Hs00271467_m1 | 6.90 | 3.42 |  | 7.41 | 3.23 | -0.51 | 1.42 | 0.6999 |  | 2.10 | 2.55 |  | 3.60 | 3.75 | -1.50 | 2.83 | 0.273 |  | 0.43 | 1.34 |  | 2.64 | 3.88 | -2.22 | 4.65 | 0.0945 |
| G02 | IFI6 | Hs00242571_m1 | 4.55 | 2.25 |  | 5.42 | 2.23 | -0.88 | 1.84 | 0.3277 |  | 2.35 | 1.58 |  | 4.69 | 2.18 | -2.35 | 5.09 | 0.0081 |  | 2.14 | 1.57 |  | 2.82 | 2.05 | -0.68 | 1.60 | 0.3399 |
| G03 | IFIT1 | Hs01675197_m1 | 8.16 | 1.99 |  | 8.83 | 1.56 | -0.67 | 1.59 | 0.3601 |  | 6.40 | 1.57 |  | 8.59 | 1.70 | -2.18 | 4.54 | 0.007 |  | 5.77 | 0.92 |  | 7.42 | 1.77 | -1.65 | 3.15 | 0.0132 |
| G04 | IFIT3 | Hs00382744_m1 | 4.77 | 1.68 |  | 5.18 | 1.26 | -0.41 | 1.33 | 0.4943 |  | 3.63 | 1.27 |  | 5.26 | 1.42 | -1.63 | 3.09 | 0.0124 |  | 2.98 | 0.62 |  | 4.35 | 1.53 | -1.37 | 2.59 | 0.015 |
| G05 | OAS1 | Hs00973637_m1 | 1.83 | 1.40 |  | 2.12 | 1.18 | -0.29 | 1.22 | 0.5757 |  | 1.48 | 0.84 |  | 2.30 | 0.72 | -0.82 | 1.76 | 0.0366 |  | 0.83 | 0.56 |  | 1.89 | 0.84 | -1.07 | 2.09 | 0.0006 |
| G06 | OAS3 | Hs00196324_m1 | 3.30 | 1.64 |  | 3.56 | 1.32 | -0.26 | 1.20 | 0.6689 |  | 2.82 | 1.18 |  | 3.86 | 1.77 | -1.04 | 2.06 | 0.1095 |  | 2.10 | 0.99 |  | 3.59 | 1.44 | -1.49 | 2.81 | 0.0038 |
| G07 | OASL | Hs00984390_m1 | 3.07 | 1.54 |  | 3.66 | 1.07 | -0.59 | 1.51 | 0.2819 |  | 2.90 | 1.14 |  | 3.83 | 1.00 | -0.94 | 1.91 | 0.0736 |  | 1.89 | 0.91 |  | 3.54 | 1.13 | -1.64 | 3.13 | 0.0003 |
| G08 | DHX58 | Hs00225561_m1 | 7.13 | 0.99 |  | 7.21 | 0.95 | -0.08 | 1.06 | 0.8346 |  | 5.71 | 0.88 |  | 6.82 | 1.17 | -1.11 | 2.16 | 0.0201 |  | 5.65 | 0.72 |  | 6.12 | 1.04 | -0.47 | 1.39 | 0.1753 |
| G09 | RSAD2 | Hs00369813_m1 | 4.41 | 2.06 |  | 4.94 | 1.67 | -0.52 | 1.44 | 0.4924 |  | 1.78 | 1.18 |  | 4.33 | 1.22 | -2.54 | 5.83 | 0.0001 |  | 1.45 | 0.95 |  | 2.89 | 1.92 | -1.45 | 2.73 | 0.0371 |
| G10 | LOC26010 | Hs00274702_m1 | 7.22 | 1.46 |  | 7.73 | 1.27 | -0.52 | 1.43 | 0.353 |  | 6.11 | 0.97 |  | 7.64 | 1.08 | -1.52 | 2.88 | 0.003 |  | 5.49 | 0.81 |  | 6.84 | 1.34 | -1.36 | 2.56 | 0.0089 |
| G11 | SIGLEC1 | Hs00988063_m1 | 3.82 | 2.78 |  | 4.72 | 2.95 | -0.90 | 1.86 | 0.4299 |  | 2.55 | 1.93 |  | 4.09 | 2.75 | -1.54 | 2.91 | 0.2129 |  | 1.68 | 1.78 |  | 2.97 | 2.00 | -1.29 | 2.44 | 0.0917 |
| G12 | MX1 | Hs00182073_m1 | 0.26 | 1.88 |  | 0.80 | 1.75 | -0.55 | 1.46 | 0.4527 |  | -0.20 | 1.24 |  | 1.00 | 1.23 | -1.20 | 2.30 | 0.0442 |  | -1.19 | 1.06 |  | 0.48 | 1.17 | -1.67 | 3.19 | 0.0007 |
| G13 | HESX1 | Hs00172696_m1 | 10.51 | 1.96 |  | 11.34 | 1.69 | -0.82 | 1.77 | 0.2687 |  | 9.57 | 1.72 |  | 10.87 | 1.54 | -1.30 | 2.46 | 0.1213 |  | 8.34 | 1.21 |  | 10.51 | 1.80 | -2.17 | 4.50 | 0.0009 |
| G14 | LAMP3 | Hs00180880_m1 | 9.02 | 1.48 |  | 8.85 | 1.58 | 0.17 | 0.89 | 0.7728 |  | 6.60 | 1.38 |  | 8.05 | 1.46 | -1.44 | 2.72 | 0.0343 |  | 6.30 | 1.22 |  | 7.01 | 1.67 | -0.70 | 1.63 | 0.2484 |
| G15 | HERC6 | Hs00215555_m1 | 4.78 | 1.45 |  | 5.16 | 1.09 | -0.38 | 1.30 | 0.4729 |  | 2.64 | 1.05 |  | 4.45 | 1.20 | -1.81 | 3.50 | 0.0015 |  | 2.43 | 0.96 |  | 3.40 | 1.38 | -0.97 | 1.96 | 0.0407 |
| G16 | HERC5 | Hs00180943_m1 | 3.93 | 1.50 |  | 4.27 | 1.16 | -0.35 | 1.27 | 0.5236 |  | 2.74 | 0.96 |  | 4.42 | 1.00 | -1.68 | 3.21 | 0.001 |  | 2.34 | 0.89 |  | 3.50 | 1.28 | -1.16 | 2.24 | 0.01 |
| G17 | ISG15 | Hs00192713_m1 | 5.14 | 2.73 |  | 6.35 | 2.56 | -1.21 | 2.31 | 0.2589 |  | 4.90 | 1.75 |  | 7.08 | 2.31 | -2.18 | 4.53 | 0.0209 |  | 3.87 | 1.98 |  | 5.86 | 1.97 | -1.99 | 3.98 | 0.0163 |
| G18 | IFI44 | Hs00197427_m1 | 2.22 | 1.50 |  | 2.69 | 1.73 | -0.47 | 1.39 | 0.4561 |  | 1.45 | 1.07 |  | 2.91 | 1.58 | -1.46 | 2.76 | 0.0166 |  | 0.73 | 0.79 |  | 2.37 | 1.53 | -1.64 | 3.12 | 0.0012 |
| G19 | IFIT2 | Hs00533665_m1 | 2.34 | 1.63 |  | 2.89 | 0.98 | -0.55 | 1.46 | 0.3312 |  | 2.68 | 1.25 |  | 3.95 | 1.22 | -1.27 | 2.42 | 0.0347 |  | 1.63 | 1.11 |  | 3.53 | 1.03 | -1.90 | 3.73 | 0.0001 |
| G20 | IFIT5 | Hs00202721_m1 | 6.07 | 1.92 |  | 6.74 | 1.72 | -0.67 | 1.59 | 0.362 |  | 4.44 | 1.61 |  | 7.03 | 2.21 | -2.59 | 6.00 | 0.0047 |  | 4.65 | 2.33 |  | 5.30 | 2.61 | -0.65 | 1.57 | 0.503 |
| G21 | SERPING1 | Hs00934330_m1 | 4.68 | 1.64 |  | 5.34 | 1.47 | -0.66 | 1.58 | 0.2936 |  | 3.28 | 1.29 |  | 5.13 | 1.42 | -1.85 | 3.61 | 0.0056 |  | 3.11 | 0.95 |  | 4.07 | 1.14 | -0.95 | 1.94 | 0.0258 |
| G22 | IFITM3 | Hs03057129_s1 | -1.92 | 1.61 |  | -1.34 | 1.28 | -0.57 | 1.49 | 0.3333 |  | -3.29 | 0.99 |  | -2.11 | 1.10 | -1.18 | 2.26 | 0.0192 |  | -3.73 | 0.82 |  | -2.55 | 1.29 | -1.18 | 2.26 | 0.0076 |
| G23 | EPSTI1 | Hs01566789_m1 | 0.49 | 1.75 |  | 1.00 | 1.66 | -0.51 | 1.42 | 0.4563 |  | -0.04 | 0.97 |  | 1.05 | 1.23 | -1.09 | 2.12 | 0.0332 |  | -0.61 | 1.00 |  | 0.42 | 1.08 | -1.03 | 2.05 | 0.0171 |
| G24 | CCL2 | Hs00234140_m1 | 6.58 | 2.32 |  | 7.64 | 2.15 | -1.06 | 2.09 | 0.2408 |  | 5.80 | 2.04 |  | 8.22 | 2.20 | -2.41 | 5.33 | 0.0187 |  | 4.38 | 1.70 |  | 7.01 | 1.90 | -2.62 | 6.17 | 0.0009 |
| G25 | CCL8 | Hs00271615_m1 | 7.77 | 2.33 |  | 8.93 | 1.92 | -1.17 | 2.24 | 0.1831 |  | 8.45 | 2.60 |  | 10.10 | 1.30 | -1.65 | 3.14 | 0.1285 |  | 6.85 | 1.79 |  | 9.60 | 1.31 | -2.75 | 6.71 | 0.0002 |
| G26 | JUP | Hs00158408_m1 | 4.45 | 2.27 |  | 5.00 | 1.92 | -0.55 | 1.47 | 0.5164 |  | 2.63 | 1.82 |  | 4.14 | 2.31 | -1.52 | 2.86 | 0.1051 |  | 2.33 | 1.63 |  | 3.08 | 1.76 | -0.76 | 1.69 | 0.2623 |
| G27 | CD300E | Hs00417308_m1 | 2.19 | 0.77 |  | 2.08 | 0.80 | 0.11 | 0.93 | 0.7313 |  | 1.15 | 0.91 |  | 2.19 | 1.45 | -1.03 | 2.04 | 0.0496 |  | 1.27 | 1.07 |  | 1.64 | 1.12 | -0.38 | 1.30 | 0.3857 |
| G28 | LGALS3BP | Hs01003086_m1 | 4.45 | 2.65 |  | 4.94 | 2.90 | -0.49 | 1.41 | 0.6522 |  | 3.06 | 1.96 |  | 4.85 | 2.94 | -1.78 | 3.44 | 0.0994 |  | 2.33 | 2.25 |  | 3.40 | 2.02 | -1.07 | 2.09 | 0.2197 |
| G29 | FFAR2 | Hs00271142_s1 | 5.77 | 2.04 |  | 6.09 | 0.99 | -0.33 | 1.25 | 0.6296 |  | 5.89 | 1.77 |  | 6.22 | 1.15 | -0.33 | 1.26 | 0.6543 |  | 4.87 | 1.22 |  | 6.21 | 1.08 | -1.33 | 2.52 | 0.0073 |
| G30 | EIF2AK2 | Hs00169345_m1 | 2.65 | 1.34 |  | 2.97 | 0.93 | -0.32 | 1.24 | 0.5041 |  | 2.18 | 0.95 |  | 3.03 | 0.85 | -0.86 | 1.81 | 0.0532 |  | 1.42 | 0.64 |  | 2.62 | 1.01 | -1.20 | 2.30 | 0.0031 |
| G31 | PNPT1 | Hs00396733_g1 | 5.01 | 1.10 |  | 5.24 | 0.83 | -0.23 | 1.17 | 0.5594 |  | 5.65 | 0.90 |  | 6.11 | 0.84 | -0.46 | 1.38 | 0.2634 |  | 4.79 | 0.96 |  | 5.90 | 0.63 | -1.12 | 2.17 | 0.0023 |
| G32 | IFIH1 | Hs01070332_m1 | 5.27 | 1.10 |  | 5.46 | 0.80 | -0.19 | 1.14 | 0.6318 |  | 3.73 | 0.82 |  | 5.17 | 0.77 | -1.43 | 2.70 | 0.0007 |  | 3.57 | 0.85 |  | 4.34 | 1.10 | -0.77 | 1.70 | 0.0506 |
| G33 | PGAP1 | Hs01088726_m1 | 10.14 | 1.16 |  | 10.27 | 0.73 | -0.14 | 1.10 | 0.7105 |  | 9.11 | 0.97 |  | 10.07 | 0.84 | -0.96 | 1.95 | 0.033 |  | 8.94 | 0.96 |  | 9.21 | 0.79 | -0.27 | 1.21 | 0.4448 |
| G34 | MX2 | Hs01550808_m1 | 0.75 | 1.43 |  | 1.05 | 1.34 | -0.30 | 1.23 | 0.5893 |  | -0.21 | 1.02 |  | 0.89 | 0.99 | -1.10 | 2.14 | 0.0261 |  | -0.68 | 0.67 |  | 0.35 | 1.11 | -1.03 | 2.04 | 0.015 |
| G35 | USP18 | Hs00276441_m1 | 5.48 | 1.83 |  | 5.46 | 1.55 | 0.02 | 0.99 | 0.9805 |  | 4.22 | 1.32 |  | 5.23 | 1.40 | -1.01 | 2.01 | 0.1125 |  | 3.15 | 0.62 |  | 4.60 | 1.43 | -1.45 | 2.74 | 0.0078 |
| G36 | APOBEC3A | Hs00377444_m1 | 3.07 | 1.78 |  | 3.41 | 1.38 | -0.34 | 1.27 | 0.5971 |  | 2.70 | 1.37 |  | 3.63 | 1.14 | -0.93 | 1.91 | 0.1322 |  | 1.72 | 0.94 |  | 3.17 | 1.02 | -1.44 | 2.72 | 0.0009 |
| G37 | PLSCR1 | Hs00275514_m1 | 1.03 | 1.20 |  | 1.49 | 1.02 | -0.47 | 1.38 | 0.3037 |  | 1.07 | 0.85 |  | 1.85 | 0.98 | -0.78 | 1.71 | 0.0671 |  | 0.38 | 0.91 |  | 1.62 | 0.88 | -1.24 | 2.37 | 0.0016 |
| G38 | DDX60 | Hs00214153_m1 | 3.23 | 1.29 |  | 3.62 | 0.98 | -0.39 | 1.31 | 0.402 |  | 2.14 | 0.84 |  | 3.34 | 0.91 | -1.19 | 2.29 | 0.006 |  | 1.72 | 0.80 |  | 2.50 | 1.12 | -0.78 | 1.72 | 0.0443 |
| G39 | CTSL1 | Hs00377632_m1 | 3.32 | 2.97 |  | 3.31 | 0.95 | 0.01 | 0.99 | 0.9907 |  | 2.38 | 0.71 |  | 3.34 | 1.11 | -0.95 | 1.94 | 0.0211 |  | 2.23 | 0.82 |  | 2.62 | 0.88 | -0.40 | 1.32 | 0.2435 |
| G40 | PPBP | Hs00234077_m1 | 0.61 | 2.70 |  | 1.64 | 2.16 | -1.03 | 2.04 | 0.3028 |  | 1.07 | 1.39 |  | 1.63 | 1.56 | -0.57 | 1.48 | 0.3945 |  | 1.85 | 1.99 |  | 1.48 | 1.52 | 0.37 | 0.77 | 0.6065 |
| G41 | GNG11 | Hs00914578_m1 | 5.12 | 2.67 |  | 5.91 | 2.29 | -0.79 | 1.73 | 0.4318 |  | 5.12 | 1.29 |  | 5.80 | 1.52 | -0.68 | 1.60 | 0.2808 |  | 5.27 | 1.42 |  | 5.23 | 1.50 | 0.04 | 0.97 | 0.9383 |
| G42 | ACAA1 | Hs01576070_m1 | 4.11 | 2.96 |  | 3.96 | 0.55 | 0.15 | 0.90 | 0.8722 |  | 4.55 | 0.48 |  | 4.44 | 0.45 | 0.10 | 0.93 | 0.6288 |  | 4.30 | 0.79 |  | 4.50 | 0.24 | -0.20 | 1.15 | 0.4188 |
| G43 | FCER1A | Hs00758600_m1 | 2.71 | 2.71 |  | 3.14 | 0.94 | -0.43 | 1.35 | 0.6178 |  | 4.08 | 0.86 |  | 4.73 | 1.80 | -0.65 | 1.57 | 0.2479 |  | 4.41 | 1.60 |  | 3.87 | 0.90 | 0.54 | 0.69 | 0.3201 |
| Endogenous control: GADPH | | |  |  |  |  |  |  |  |  |  |  |  |  |  |  |  |  |  |  |  |  |  |  |  |  |  |  |

| Supplementary table 4. Comparison of the expression of genes between RVR and non-RVR | | | | | | | | | |  |  |  |  |  |  |  |  |  |  |  |  |  |  |  |  |  |  |  |
| --- | --- | --- | --- | --- | --- | --- | --- | --- | --- | --- | --- | --- | --- | --- | --- | --- | --- | --- | --- | --- | --- | --- | --- | --- | --- | --- | --- | --- |
|  |  |  | Week 0 |  |  |  |  |  |  |  |  | Week 1 |  |  |  |  |  |  |  |  | Week 4 |  |  |  |  |  |  |  |
|  | dCT |  | RVR (n=14) | |  | non-RVR (n=13) | |  |  |  |  | RVR (n=14) | |  | non-RVR (n=9) | |  |  |  |  | RVR (n=14) | |  | non-RVR (n=13) | |  |  |  |
|  | Gene | Accession no. | Mean | SD |  | Mean | SD | ddCt | Fold | p-value |  | Mean | SD |  | Mean | SD | ddCt | Fold | p-value |  | Mean | SD |  | Mean | SD | ddCt | Fold | p-value |
| G01 | IFI27 | Hs00271467_m1 | 6.53 | 3.13 |  | 7.72 | 3.47 | -1.18 | 2.27 | 0.3598 |  | 1.99 | 2.76 |  | 3.43 | 3.22 | -1.44 | 2.71 | 0.2652 |  | 0.31 | 1.25 |  | 2.43 | 3.64 | -2.12 | 4.35 | 0.0651 |
| G02 | IFI6 | Hs00242571_m1 | 4.13 | 1.48 |  | 5.74 | 2.65 | -1.61 | 3.06 | 0.069 |  | 2.22 | 1.60 |  | 4.37 | 2.06 | -2.15 | 4.45 | 0.0104 |  | 1.79 | 1.30 |  | 3.10 | 2.00 | -1.31 | 2.48 | 0.0526 |
| G03 | IFIT1 | Hs01675197_m1 | 8.00 | 1.85 |  | 8.91 | 1.75 | -0.91 | 1.88 | 0.2003 |  | 6.46 | 1.71 |  | 8.01 | 1.81 | -1.55 | 2.94 | 0.0504 |  | 5.60 | 0.83 |  | 7.34 | 1.65 | -1.74 | 3.34 | 0.0031 |
| G04 | IFIT3 | Hs00382744_m1 | 4.60 | 1.61 |  | 5.30 | 1.35 | -0.70 | 1.62 | 0.2372 |  | 3.68 | 1.33 |  | 4.82 | 1.55 | -1.14 | 2.20 | 0.074 |  | 2.85 | 0.52 |  | 4.27 | 1.42 | -1.42 | 2.68 | 0.004 |
| G05 | OAS1 | Hs00973637_m1 | 1.66 | 1.17 |  | 2.25 | 1.40 | -0.60 | 1.51 | 0.2396 |  | 1.46 | 0.90 |  | 2.15 | 0.70 | -0.69 | 1.62 | 0.0636 |  | 0.78 | 0.55 |  | 1.79 | 0.84 | -1.01 | 2.02 | 0.001 |
| G06 | OAS3 | Hs00196324_m1 | 2.96 | 1.54 |  | 3.89 | 1.34 | -0.93 | 1.90 | 0.1098 |  | 2.78 | 1.26 |  | 3.68 | 1.58 | -0.89 | 1.86 | 0.1465 |  | 1.92 | 0.86 |  | 3.56 | 1.36 | -1.64 | 3.12 | 0.0009 |
| G07 | OASL | Hs00984390_m1 | 2.88 | 1.46 |  | 3.78 | 1.16 | -0.90 | 1.87 | 0.0896 |  | 2.81 | 1.17 |  | 3.76 | 0.93 | -0.95 | 1.93 | 0.0525 |  | 1.83 | 0.90 |  | 3.35 | 1.18 | -1.52 | 2.87 | 0.0009 |
| G08 | DHX58 | Hs00225561_m1 | 7.02 | 0.91 |  | 7.31 | 1.01 | -0.29 | 1.23 | 0.4363 |  | 5.87 | 1.03 |  | 6.33 | 1.16 | -0.46 | 1.37 | 0.3331 |  | 5.56 | 0.74 |  | 6.14 | 0.94 | -0.58 | 1.50 | 0.0849 |
| G09 | RSAD2 | Hs00369813_m1 | 4.17 | 1.91 |  | 5.12 | 1.82 | -0.96 | 1.94 | 0.1945 |  | 1.81 | 1.33 |  | 3.72 | 1.51 | -1.91 | 3.75 | 0.0044 |  | 1.21 | 0.84 |  | 2.93 | 1.71 | -1.72 | 3.29 | 0.0025 |
| G10 | LOC26010 | Hs00274702_m1 | 6.98 | 1.37 |  | 7.91 | 1.28 | -0.93 | 1.90 | 0.0818 |  | 6.11 | 1.01 |  | 7.31 | 1.18 | -1.21 | 2.31 | 0.016 |  | 5.32 | 0.72 |  | 6.82 | 1.22 | -1.50 | 2.84 | 0.0006 |
| G11 | SIGLEC1 | Hs00988063_m1 | 3.05 | 1.99 |  | 5.41 | 3.16 | -2.36 | 5.12 | 0.0275 |  | 2.30 | 1.87 |  | 4.13 | 2.47 | -1.83 | 3.55 | 0.0569 |  | 1.32 | 1.34 |  | 3.16 | 2.09 | -1.84 | 3.58 | 0.0135 |
| G12 | MX1 | Hs00182073_m1 | -0.11 | 1.65 |  | 1.11 | 1.83 | -1.21 | 2.32 | 0.0824 |  | -0.34 | 1.25 |  | 0.96 | 1.08 | -1.31 | 2.48 | 0.0178 |  | -1.35 | 0.94 |  | 0.39 | 1.19 | -1.74 | 3.34 | 0.0003 |
| G13 | HESX1 | Hs00172696_m1 | 10.45 | 1.85 |  | 11.28 | 1.86 | -0.84 | 1.78 | 0.2533 |  | 9.53 | 1.71 |  | 10.61 | 1.68 | -1.08 | 2.11 | 0.1675 |  | 8.34 | 1.04 |  | 10.17 | 2.01 | -1.83 | 3.56 | 0.0059 |
| G14 | LAMP3 | Hs00180880_m1 | 8.92 | 1.53 |  | 8.98 | 1.51 | -0.05 | 1.04 | 0.926 |  | 6.82 | 1.58 |  | 7.39 | 1.47 | -0.57 | 1.49 | 0.3958 |  | 6.26 | 1.30 |  | 6.94 | 1.53 | -0.68 | 1.61 | 0.2214 |
| G15 | HERC6 | Hs00215555_m1 | 4.66 | 1.45 |  | 5.23 | 1.12 | -0.57 | 1.48 | 0.2661 |  | 2.78 | 1.14 |  | 3.83 | 1.50 | -1.05 | 2.06 | 0.0717 |  | 2.27 | 0.94 |  | 3.43 | 1.23 | -1.16 | 2.24 | 0.0105 |
| G16 | HERC5 | Hs00180943_m1 | 3.69 | 1.43 |  | 4.47 | 1.21 | -0.78 | 1.72 | 0.1403 |  | 2.78 | 0.95 |  | 3.99 | 1.31 | -1.21 | 2.32 | 0.0174 |  | 2.15 | 0.80 |  | 3.53 | 1.15 | -1.39 | 2.61 | 0.0012 |
| G17 | ISG15 | Hs00192713_m1 | 4.74 | 2.23 |  | 6.59 | 2.88 | -1.85 | 3.61 | 0.072 |  | 4.59 | 1.71 |  | 7.08 | 1.90 | -2.49 | 5.63 | 0.0037 |  | 3.40 | 1.56 |  | 6.06 | 1.92 | -2.66 | 6.30 | 0.0006 |
| G18 | IFI44 | Hs00197427_m1 | 2.25 | 1.46 |  | 2.58 | 1.75 | -0.33 | 1.25 | 0.6017 |  | 1.42 | 1.09 |  | 2.63 | 1.55 | -1.22 | 2.32 | 0.0381 |  | 0.63 | 0.71 |  | 2.22 | 1.49 | -1.59 | 3.01 | 0.0015 |
| G19 | IFIT2 | Hs00533665_m1 | 2.17 | 1.60 |  | 2.99 | 1.07 | -0.82 | 1.77 | 0.131 |  | 2.62 | 1.26 |  | 3.77 | 1.26 | -1.14 | 2.21 | 0.0451 |  | 1.54 | 1.09 |  | 3.33 | 1.14 | -1.78 | 3.45 | 0.0003 |
| G20 | IFIT5 | Hs00202721_m1 | 5.74 | 1.60 |  | 6.99 | 1.91 | -1.25 | 2.38 | 0.0759 |  | 4.41 | 1.72 |  | 6.50 | 2.19 | -2.09 | 4.26 | 0.0184 |  | 4.00 | 1.82 |  | 5.91 | 2.65 | -1.91 | 3.77 | 0.0373 |
| G21 | SERPING1 | Hs00934330_m1 | 4.35 | 1.55 |  | 5.59 | 1.39 | -1.25 | 2.37 | 0.0381 |  | 3.20 | 1.32 |  | 4.84 | 1.43 | -1.63 | 3.11 | 0.0106 |  | 2.92 | 0.78 |  | 4.13 | 1.11 | -1.20 | 2.30 | 0.0029 |
| G22 | IFITM3 | Hs03057129_s1 | -2.15 | 1.48 |  | -1.18 | 1.36 | -0.98 | 1.97 | 0.0872 |  | -3.38 | 1.01 |  | -2.24 | 1.03 | -1.14 | 2.20 | 0.0164 |  | -3.91 | 0.57 |  | -2.54 | 1.25 | -1.37 | 2.59 | 0.0022 |
| G23 | EPSTI1 | Hs01566789_m1 | 0.15 | 1.43 |  | 1.29 | 1.82 | -1.13 | 2.19 | 0.0827 |  | -0.24 | 0.91 |  | 1.12 | 1.00 | -1.36 | 2.57 | 0.0029 |  | -0.84 | 0.84 |  | 0.51 | 1.01 | -1.35 | 2.55 | 0.0008 |
| G24 | CCL2 | Hs00234140_m1 | 6.64 | 2.27 |  | 7.41 | 2.30 | -0.77 | 1.70 | 0.3908 |  | 5.71 | 2.08 |  | 7.82 | 2.22 | -2.11 | 4.31 | 0.0312 |  | 4.30 | 1.32 |  | 6.70 | 2.29 | -2.40 | 5.29 | 0.0037 |
| G25 | CCL8 | Hs00271615_m1 | 7.75 | 2.27 |  | 8.76 | 2.10 | -1.01 | 2.02 | 0.242 |  | 8.16 | 2.31 |  | 10.18 | 2.03 | -2.02 | 4.06 | 0.0439 |  | 6.84 | 1.34 |  | 9.19 | 2.12 | -2.35 | 5.09 | 0.0019 |
| G26 | JUP | Hs00158408_m1 | 3.77 | 1.36 |  | 5.65 | 2.39 | -1.88 | 3.67 | 0.0226 |  | 2.34 | 1.59 |  | 4.25 | 2.25 | -1.91 | 3.75 | 0.0265 |  | 1.89 | 1.10 |  | 3.44 | 1.88 | -1.54 | 2.91 | 0.0147 |
| G27 | CD300E | Hs00417308_m1 | 1.94 | 0.78 |  | 2.37 | 0.73 | -0.43 | 1.35 | 0.1495 |  | 1.02 | 0.91 |  | 2.16 | 1.25 | -1.14 | 2.20 | 0.0193 |  | 0.94 | 0.75 |  | 1.94 | 1.17 | -0.99 | 1.99 | 0.014 |
| G28 | LGALS3BP | Hs01003086_m1 | 3.77 | 1.97 |  | 5.58 | 3.15 | -1.81 | 3.51 | 0.0912 |  | 2.68 | 1.69 |  | 5.04 | 2.67 | -2.36 | 5.13 | 0.0166 |  | 1.72 | 1.59 |  | 3.89 | 2.23 | -2.16 | 4.48 | 0.0073 |
| G29 | FFAR2 | Hs00271142_s1 | 5.49 | 2.09 |  | 6.34 | 0.98 | -0.84 | 1.80 | 0.1965 |  | 6.04 | 1.95 |  | 5.92 | 0.86 | 0.12 | 0.92 | 0.8645 |  | 4.89 | 1.38 |  | 5.98 | 1.04 | -1.09 | 2.12 | 0.0303 |
| G30 | EIF2AK2 | Hs00169345_m1 | 2.48 | 1.33 |  | 3.10 | 0.94 | -0.63 | 1.54 | 0.1722 |  | 2.21 | 0.97 |  | 2.79 | 0.97 | -0.57 | 1.49 | 0.1808 |  | 1.34 | 0.61 |  | 2.52 | 0.98 | -1.18 | 2.26 | 0.0008 |
| G31 | PNPT1 | Hs00396733_g1 | 4.95 | 1.16 |  | 5.27 | 0.78 | -0.32 | 1.25 | 0.4096 |  | 5.61 | 0.85 |  | 6.08 | 0.92 | -0.47 | 1.38 | 0.2273 |  | 4.75 | 0.93 |  | 5.76 | 0.80 | -1.01 | 2.02 | 0.0057 |
| G32 | IFIH1 | Hs01070332_m1 | 5.11 | 1.08 |  | 5.60 | 0.81 | -0.50 | 1.41 | 0.1905 |  | 3.79 | 0.91 |  | 4.76 | 0.98 | -0.96 | 1.95 | 0.0254 |  | 3.34 | 0.72 |  | 4.47 | 0.97 | -1.13 | 2.19 | 0.002 |
| G33 | PGAP1 | Hs01088726_m1 | 10.03 | 1.20 |  | 10.37 | 0.71 | -0.34 | 1.26 | 0.3823 |  | 9.30 | 1.03 |  | 9.57 | 1.03 | -0.27 | 1.21 | 0.5452 |  | 8.76 | 0.85 |  | 9.37 | 0.85 | -0.61 | 1.53 | 0.073 |
| G34 | MX2 | Hs01550808_m1 | 0.47 | 1.25 |  | 1.30 | 1.42 | -0.83 | 1.78 | 0.1193 |  | -0.29 | 1.05 |  | 0.78 | 0.91 | -1.08 | 2.11 | 0.0199 |  | -0.85 | 0.54 |  | 0.37 | 1.01 | -1.21 | 2.32 | 0.0012 |
| G35 | USP18 | Hs00276441_m1 | 5.36 | 1.71 |  | 5.60 | 1.73 | -0.23 | 1.18 | 0.7277 |  | 4.23 | 1.42 |  | 5.00 | 1.29 | -0.76 | 1.70 | 0.2077 |  | 3.12 | 0.63 |  | 4.41 | 1.40 | -1.29 | 2.45 | 0.0075 |
| G36 | APOBEC3A | Hs00377444_m1 | 2.78 | 1.57 |  | 3.68 | 1.56 | -0.90 | 1.87 | 0.1484 |  | 2.55 | 1.38 |  | 3.67 | 1.05 | -1.12 | 2.17 | 0.0506 |  | 1.63 | 0.81 |  | 3.05 | 1.12 | -1.42 | 2.68 | 0.0009 |
| G37 | PLSCR1 | Hs00275514_m1 | 0.88 | 1.15 |  | 1.57 | 1.04 | -0.69 | 1.61 | 0.1165 |  | 0.96 | 0.82 |  | 1.84 | 0.90 | -0.88 | 1.84 | 0.0255 |  | 0.19 | 0.74 |  | 1.63 | 0.88 | -1.44 | 2.71 | 0.0001 |
| G38 | DDX60 | Hs00214153_m1 | 2.99 | 1.06 |  | 3.81 | 1.17 | -0.82 | 1.77 | 0.0666 |  | 2.09 | 0.85 |  | 3.15 | 0.93 | -1.06 | 2.08 | 0.0108 |  | 1.53 | 0.74 |  | 2.59 | 0.98 | -1.05 | 2.08 | 0.0041 |
| G39 | CTSL1 | Hs00377632_m1 | 3.28 | 1.29 |  | 3.36 | 3.16 | -0.09 | 1.06 | 0.9267 |  | 2.29 | 0.71 |  | 3.27 | 0.97 | -0.98 | 1.97 | 0.0107 |  | 2.06 | 0.76 |  | 2.74 | 0.84 | -0.68 | 1.60 | 0.0377 |
| G40 | PPBP | Hs00234077_m1 | 0.62 | 1.42 |  | 1.48 | 3.31 | -0.85 | 1.81 | 0.3864 |  | 1.06 | 1.38 |  | 1.52 | 1.54 | -0.47 | 1.38 | 0.4584 |  | 1.52 | 1.32 |  | 1.90 | 2.24 | -0.38 | 1.30 | 0.5916 |
| G41 | GNG11 | Hs00914578_m1 | 5.29 | 1.56 |  | 5.61 | 3.30 | -0.32 | 1.25 | 0.7457 |  | 5.16 | 1.27 |  | 5.60 | 1.54 | -0.44 | 1.36 | 0.4635 |  | 5.21 | 1.38 |  | 5.29 | 1.53 | -0.08 | 1.06 | 0.884 |
| G42 | ACAA1 | Hs01576070_m1 | 4.04 | 1.21 |  | 4.06 | 3.11 | -0.02 | 1.01 | 0.9831 |  | 4.55 | 0.48 |  | 4.47 | 0.45 | 0.07 | 0.95 | 0.7254 |  | 4.16 | 0.52 |  | 4.63 | 0.66 | -0.47 | 1.38 | 0.0499 |
| G43 | FCER1A | Hs00758600_m1 | 2.66 | 1.29 |  | 3.13 | 2.85 | -0.46 | 1.38 | 0.5864 |  | 3.96 | 0.81 |  | 4.78 | 1.59 | -0.83 | 1.77 | 0.1135 |  | 4.41 | 1.67 |  | 3.96 | 0.94 | 0.45 | 0.73 | 0.3973 |
| Endogenous control: GADPH | | |  |  |  |  |  |  |  |  |  |  |  |  |  |  |  |  |  |  |  |  |  |  |  |  |  |  |
